# First activity and interactions in thalamus and cortex using raw single-trial EEG and MEG elicited by somatosensory stimulation

**DOI:** 10.3389/fnsys.2023.1305022

**Published:** 2024-01-05

**Authors:** Christodoulos Karittevlis, Michail Papadopoulos, Vinicius Lima, Gregoris A. Orphanides, Shubham Tiwari, Marios Antonakakis, Vicky Papadopoulou Lesta, Andreas A. Ioannides

**Affiliations:** ^1^AAI Scientific Cultural Services Ltd., Nicosia, Cyprus; ^2^Department of Computer Science, European University Cyprus, Nicosia, Cyprus; ^3^Aix Marseille Université, INSERM, Institut de Neurosciences des Systèmes, Marseille, France; ^4^Barts and The London School of Medicine and Dentistry, Queen Mary University of London, London, United Kingdom; ^5^Department of Geography, Durham University, Durham, United Kingdom; ^6^School of Electrical and Computer Engineering, Technical University of Crete, Chania, Greece; ^7^Institute for Biomagnetism and Biosignal Analysis, Medicine Faculty, University of Münster, Münster, Germany

**Keywords:** electroencephalography, magnetoencephalography, somatosensory, thalamocortical connectivity, spatial filtering, clustering, single-trial analysis

## Abstract

**Introduction:**

One of the primary motivations for studying the human brain is to comprehend how external sensory input is processed and ultimately perceived by the brain. A good understanding of these processes can promote the identification of biomarkers for the diagnosis of various neurological disorders; it can also provide ways of evaluating therapeutic techniques. In this work, we seek the minimal requirements for identifying key stages of activity in the brain elicited by median nerve stimulation.

**Methods:**

We have used a priori knowledge and applied a simple, linear, spatial filter on the electroencephalography and magnetoencephalography signals to identify the early responses in the thalamus and cortex evoked by short electrical stimulation of the median nerve at the wrist. The spatial filter is defined first from the average EEG and MEG signals and then refined using consistency selection rules across ST. The refined spatial filter is then applied to extract the timecourses of each ST in each targeted generator. These ST timecourses are studied through clustering to quantify the ST variability. The nature of ST connectivity between thalamic and cortical generators is then studied within each identified cluster using linear and non-linear algorithms with time delays to extract linked and directional activities. A novel combination of linear and non-linear methods provides in addition discrimination of influences as excitatory or inhibitory.

**Results:**

Our method identifies two key aspects of the evoked response. Firstly, the early onset of activity in the thalamus and the somatosensory cortex, known as the P14 and P20 in EEG and the second M20 for MEG. Secondly, good estimates are obtained for the early timecourse of activity from these two areas. The results confirm the existence of variability in ST brain activations and reveal distinct and novel patterns of connectivity in different clusters.

**Discussion:**

It has been demonstrated that we can extract new insights into stimulus processing without the use of computationally costly source reconstruction techniques which require assumptions and detailed modeling of the brain. Our methodology, thanks to its simplicity and minimal computational requirements, has the potential for real-time applications such as in neurofeedback systems and brain-computer interfaces.

## 1 Introduction

The human brain consists of around 80 billion neurons of different shapes and sizes. These neurons are highly interconnected and form complex networks in the brain with information exchanged between neurons through chemical and electrical synapses (Ioannides, 2006). The electrical activity of a single neuron is too small to be detected from outside the brain. The well-known non-invasive signals of electroencephalography (EEG) and magnetoencephalography (MEG), are the result of synergistic accumulation of contributions from a huge number of activated neurons (Hämäläinen et al., 1993; Lopes da Silva, 2013; Singh, 2014). EEG and MEG are members of the wider family of neuroimaging techniques capable of mapping mass regional activity from the brain (Horwitz et al., 2000; Bandettini, 2009; He et al., 2011). These techniques can identify activity confined to a small enough region that belong to specific cytoarchitectonic areas in the cortex (Moradi et al., 2003) or specific nuclei (Papadelis et al., 2011, 2012). It is universally acknowledged that what distinguishes EEG and MEG from other neuroimaging techniques is their high temporal resolution which is in the order of a millisecond or less (Hämäläinen et al., 1993; He et al., 2011; Lopes da Silva, 2013; Baillet, 2017; Michel and He, 2019).

EEG measures the electric potential difference between the ground and other scalp surface electrodes. In the case of MEG, the instantaneous changes in the magnetic field are measured using either single coil sensors (magnetometers) or differential combination of coils (gradiometers) (Hämäläinen et al., 1993; Singh, 2014). Modern instruments provide a collection of sensors for each modality arranged uniformly to cover as much as possible the surface of the head around the brain (Baillet, 2017).

The standard way to estimate the activity inside the brain is through source reconstruction algorithms (Asadzadeh et al., 2020), which transform the timecourses of sensor recordings (signal space description) to timecourses of few or many generators in the brain (source space description) (Ioannides et al., 1990; Mosher et al., 1999; Michel and Brunet, 2019). Source reconstruction using EEG and/or MEG, demands making a model which is based on assumptions some explicit while other implicit; a model allows computations to be made for the contribution from any assumed source configuration (forward problem) and hence through applications of different methods (which also contain assumptions) a best fit solution can be determined (Scherg, 1992; Mosher et al., 1999; Michel and Brunet, 2019; Asadzadeh et al., 2020). For EEG, a complex model is necessary to take into account the high resistivity of the skull (Michel and Brunet, 2019) and the changes in conductivity between different tissues that distort the electric current (Baillet, 2017). In contrast, for MEG, a simpler model is sufficient as the propagation of the magnetic field is not greatly affected by the conductivity details (Hämäläinen et al., 1993; Lopes da Silva, 2013; Singh, 2014). Nevertheless, in both cases, the accuracy and spatial resolution depend heavily on the accurate co-registration of the sensor's position to the head (Akalin Acar and Makeig, 2013; Michel and Brunet, 2019).

There are numerous EEG/MEG studies involving animals and human subjects that aim to understand the underlying mechanisms of different sensory systems, by using different kinds of external stimulation. To understand the processing mechanisms of any sensory system, apart from identifying the location of the brain areas involved in the processing, what is also necessary is the study of the communication between those areas (He et al., 2011). During an experiment, a large number of identical stimuli are presented to the subject, with each presentation referred to as a single-trial (ST). Even though identical stimuli are used, each stimulus evokes a response that could vary considerably between trials (ST variability), in terms of the timing and topography of the EEG and MEG signal (Liu et al., 1998; Ioannides et al., 2002; Stephani et al., 2020). The interest in ST variability of EEG and MEG sensory evoked responses was raised when variability of ST responses was also demonstrated with functional magnetic resonance imaging fMRI (Duann et al., 2002). However, we will not discuss the fMRI ST variability further because the timescales involved (few seconds) are more than two orders of magnitude larger than the timescales relevant to the variability of the EEG/MEG responses (a few milliseconds).

In this paper, we utilize simultaneous EEG-MEG recordings elicited by somatosensory electrical stimulation to address the following objectives. The first objective of this study is to extract the activity of specific brain generators at particular time-points relative to the stimulus onset, while avoiding the assumptions typically associated with source reconstruction techniques. This is accomplished using Virtual Sensors (VS), which are created through linear combination of different channels. We demonstrate that such a VS can effectively capture the activity generated by generators for each ST. Combining this capability with knowledge about the location and timing of well-known generators, yields a data-driven estimate of the ST timecourse of these specific, well-known cortical and subcortical brain regions. The second objective is to verify whether or not accurate estimates of cortical and subcortical generators can indeed be extracted for each ST. The third objective of our study is to explore the usefulness of having simultaneous measurements of EEG and MEG, which are known to be derived from complementary mathematical properties of the common current density vector generated by neural electrical activity. The fourth objective is to explore the variability of the ST activations derived from the VS by utilizing clustering techniques. The fifth objective is to compute the connectivity across the ensemble of all STs using a linear and nonlinear connectivity metrics and compare the results with current literature. The sixth and final objective is to explore the variability in connectivity across STs in general and to investigate the distinct types of connectivity that prevail in the different sets of STs that clustering methods have identified.

## 2 Materials and methods

**Figure 1:**
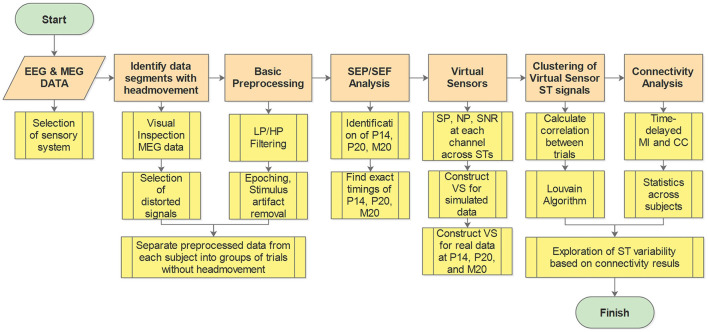
Work flow diagram. Orange boxes show the main analysis steps while the yellow boxes give a brief description of the processing and analysis methods at each step. LP, low pass; HP, high pass; SEP, somatosensory evoked potential; SEF, somatosensory evoked fields; SP, signal power; NP, noise power; SNR, signal to noise ratio; ST, single trial; VS, virtual sensor; MI, mutual information; CC, correlation coefficient.

The workflow diagram illustrating the methodology is presented in Figure 1. In the Figure, the main analysis steps are depicted in orange, while the submethods for each step are shown in yellow. A key preliminary task was to decide which sensory system to study and how this system will be stimulated. Having in mind the objectives of the study, we eventually chose to work with brain responses to somatosensory stimulation for reasons explained in Section 2.1. After this preliminary task was completed and the data obtained (Section 2.2), the first step of the pipeline involved identifying and excluding data segments contaminated with artifacts, e.g., caused by the subject's head movement during the recording (see Section 2.3). Preprocessing of the data was then conducted, which includes various filtering techniques and removal of stimulus artifacts (see Section 2.3). Subsequently, the Somatosensory Evoked Potentials (SEPs) and Fields (SEFs) were analyzed to identify the timings of the first evoked responses in the thalamus and the somatosensory cortex (see Section 2.4). Moving forward, VS were utilized to extract spatiotemporal estimations of activity in the thalamus and the primary somatosensory cortex S1 (see Section 2.5). Cluster analysis was then employed to identify and group spatiotemporal estimations with similar information (see Section 2.6). Next, the clustered data were utilized to estimate thalamocortical connectivity (see Section 2.7). Finally, the estimated connectivity values were further analyzed to differentiate the different types of influences between the thalamus and the cortex implied by the linear and non-linear connectivity measures (see Section 2.8).

### 2.1 Selection of sensory system

The EEG and MEG neuroimaging modalities are usually used for the study of the somatomotor, visual and auditory systems in the brain. Any one of these three modalities could be used in our study; the selection was influenced by how well the primary requirements, which were to have good a priori information about the timing and location of the first evoked responses in the thalamus and the cortex. For each one of these three sensory modalities (somatosensory, visual and auditory), there is good evidence in the literature about the timings and precise location of the first arrival of the responses evoked by stimuli, first in the corresponding nucleus of the thalamus [ventral posterolateral (VPL) nucleus, Lateral Geniculate nucleus (LGN) and medial geniculate body (MGB)], and a few milliseconds later to the specific primary sensory cortex [S1; Broadman Area 3b (BA3b), V1; BA17 and A1; BA41;42].

There are also common drawbacks and problems specific for each sensory modality. The common feature (drawback) is that for each modality the response to each individual stimulus is different. As demonstrated in our earlier MEG studies, for stimuli well above the sensory threshold, but relatively weak to startle the subject, the early cortical response in the primary sensory cortex is so variable that little or no evidence of it can be traced for most STs in either somatosensory (Ioannides et al., 2002; Hu et al., 2011, 2014; Stephani et al., 2020; Waterstraat et al., 2021), visual (Duann et al., 2002; Laskaris et al., 2003, 2004; Bagshaw and Warbrick, 2007) or auditory (Liu et al., 1998; Kisley and Gerstein, 1999; Laskaris and Ioannides, 2001, 2002; Goldman et al., 2009) cortex. Analysis of the ST data at signal and source levels focusing on the cortical responses has demonstrated an individual statistical signature of the evoked response that was reproducible across days (Liu et al., 1998). Also, there is a clear influence of the prestimulus activity in the primary sensory cortex on the early evoked response in the same area for somatosensory, visual and auditory stimulation (Laskaris and Ioannides, 2002; Laskaris et al., 2003). Ideally, these problems of studying ST variability can be addressed by using strong stimuli and asking the subject to report on the perceived qualities of each ST stimulation. However, such an approach is impractical because it would require long recording times and it will be very tedious and uncomfortable for the subject. It is also important to note that what we call drawback and problem above are really properties of a healthy brain, so if we want to understand normal brain function we must accept and account for the observed variability in ST responses to identical stimuli.

There are also further problems specific to each sensory modality. For visual stimulation, the placement of a stimulus to a specific quadrant of the visual field allows for a precise definition of which part of V1 the activity should be seen on the left or right hemisphere and on the upper or lower side of the calcarine fissure, provided that the subject can fixate on a fixation stimulus. No fixation is needed for auditory stimuli, but the pathway from the ear to A1 is not strictly unilateral but bilateral: auditory stimulation of one ear exiting the thalamus and the A1 on the ipsilateral and contralateral hemisphere with the pathways crossing the hemispheres at multiple subcortical levels. In contrast, the cortical sources to somatosensory stimulation are well separated and away from the mid-line of each hemisphere (for stimuli delivered in the left and right upper limbs). In addition, the reporting by the subject of the qualities of perception of each stimulus is the only way of ensuring that the targeted pathway was activated in each ST. These limitations of the visual and auditory systems (for achieving the objectives of our study) prompted us to select a fairly strong hand stimulation, i.e., electrical stimulation of the median nerve of the wrist. This type of stimulation has its own problems which we describe next in the context of other choices for exciting the somatosensory system.

Pure somatosensory stimulation can be achieved with the use of tactile stimuli, which however are difficult to control precisely (e.g., keeping the level of pressure applied constant) and/or do not afford precise timing, e.g., using a balloon. An electrical stimulation with a short pulse (sub-millisecond duration), provides an easier way of achieving precise timing. Unfortunately, the nature of electrical stimulation introduces a strong artifact, which is smaller for weak stimuli and reduces further for foot (tibial nerve) than hand electrical stimulation. Foot stimulation however is close to the top of the central sulcus with left and right primary sensory areas close to each other. Considering all factors we settled on the use of somatosensory dataset (Antonakakis et al., 2019); hereafter we will refer to electrical median nerve stimulation of the wrist as Electric Wrist (EW) stimulation, for consistency with the earlier analysis of the same data by Antonakakis and colleagues. This set includes simultaneous EEG and MEG recordings for three subjects in response to over 1,000 STs of EW stimulation at the right wrist. The EW stimulation is strong enough to produce a small twitch of the thumb. This ensures that there is a visible response for each stimulus applied, without the need to ask for a report from the subject. On the cost side, there is an early artifact in the signal caused by the strong electrical stimulus and the inevitable involvement of the motor system. A compromise of the advantage and disadvantage of the choice of a strong EW stimulation is made in the way the artifact is reduced but not completely removed as can be seen in the Supplementary Figures S2, S3. The delivery of a large number of trials takes a rather long period of time (9 min) with a stimulus that is rather slightly annoying, so some small movement could be made. While for EEG the removal of STs close to each movement is sufficient (because the electrodes are fixed on the scalp) for MEG this implies that the initial coregistration of the MEG sensors with the head is lost after the first movement.

### 2.2 Data description

We used simultaneous EEG and MEG data, collected in response to somatosensory stimulation, from three right-handed healthy human subjects. The experiments for data collection were conducted at the Institute of Biomagnetism and Biosignal Analysis, Muenster, Germany. The EEG/MEG recordings were provided to us in an anonymized form by the corresponding author of the study (Antonakakis et al., 2019) and co-author of this paper. The EEG recording involved 74 EEG channels and six auxiliary channels for eye movement detection. For MEG recording, a whole-head MEG system with 275 axial gradiometers was utilized. Further details regarding the EEG/MEG systems can be found in Antonakakis et al. (2019). In that study, three different types of stimulation were employed: Pneumato-Tactile stimulation using a balloon diaphragm, Braille-Tactile stimulation, and Electrical-Wrist (EW) stimulation of the right median nerve. For the current study, we exclusively utilized data acquired in response to EW stimulation. The electrical pulses delivered to the wrist had a duration of 0.5 ms, and the stimulus intensity was adjusted to elicit observable thumb movement in the subject. The data was sampled at a frequency of 1,200 Hz, and an online low-pass filter of 300 Hz was applied. The experiment consisted of 1,198 trials (repetitions) with an inter-stimulus interval (ISI) ranging between 350–450 ms.

### 2.3 Data preprocessing

Prior to initiating any data preprocessing, we excluded recorded periods during which large artifacts were present which are likely to involve head or body movements or large scale muscle activity like teeth clenching. Head movement during recordings significantly distorts the signals, particularly the MEG signals. Given that the MEG sensors are stationary above the head, even a small head movement causes large signal distortions across the entire sensor array that can be easily identified by visual inspection of the raw signals. Therefore, to ensure that the signals used in our analysis pipeline were free from movement artifacts, we employed a simple method to identify and exclude the trials associated with head movement. The raw signals from all MEG channels were plotted in a single plot, with each channel displayed consecutively (refer to Supplementary Figure S1). Visual inspection of the raw signals throughout the entire recording duration identified periods exhibiting significant signal distortions across all channels. These periods, along with a 1 s buffer before and after the identified noisy segments, were marked for exclusion. The STs in each period between the cut-out segments were identified as distinct set of trials, for which there was no pronounced distortion observed across all channels and hence no large head (or body) movement. This is a conservative approach because, it eliminates not only movement artifacts but also any other influence that is felt across the MEG sensor array; this conservative segmentation of the data was performed for all three subjects.

Moving to the preprocessing of the selected data, the first step was the reduction of the artifact created by the electrical stimulation, from both the EEG and MEG raw signals. For the reduction of the stimulus artifact, a segment from the background data in the pre-stimulus period (−18.3 to −5 ms) together with a segment of data containing the stimulus artifact (−5 to 8.3 ms) were multiplied by a Generalized Logistic function and combined to replace the stimulus artifact period. Detailed description of the stimulus artifact reduction method can be found in the schematic diagram shown in Supplementary Figure S2. As a second step, the EEG and MEG data were high-pass filtered at 1 Hz, and any noisy channels were removed. In the case of EEG, the mean of all channels was used to re-reference the signals of individual EEG electrodes. Furthermore, notch filtering at 50 Hz and its harmonics (100, 150, 200, 250 Hz) was applied to remove power line noise in the EEG and MEG signals. Next, the data were band pass filtered in the frequency range (20, 250 Hz). Once the data were cleaned and filtered, they were parsed into epochs (trials) of 0.3 s in total duration. Each trial consisted of signals starting 100 ms before (pre-stimulus period) and 200 ms after (post-stimulus period) stimulus onset. Subsequently, the identified trials with large movement were excluded and the remaining trials were separated into groups of trials free of movement artifacts. Finally, for each subject, the three groups with the highest number of trials were selected for further analysis. The number of trials in each group and the selected group of trials are shown for each of the three subjects in the Supplementary Table S1. This overcautious approach ensures that in each one of the groups separated out the head was in the same position for all the trials in each group.

### 2.4 Somatosensory evoked potentials/fields

While the EW stimulation excites many areas across the brain, both invasive and non-invasive experiments with humans have identified the time of the first arrival of the signal in the area of the thalamus at around 14–16 ms post-stimulus onset (Buchner et al., 1995; Hanajima, 2004; Porcaro et al., 2008; Götz et al., 2014; Politof et al., 2021). Then, the signal travels in a dorso-lateral direction reaching the primary somatosensory cortex (S1) at around 20 ms post-stimulus onset (Allison et al., 1991; Peterson et al., 1995; Gobbelé et al., 1999; Porcaro et al., 2008; Politof et al., 2019, 2021). For our purposes, what is important is that these are the first arrivals of the evoked response at the level of the thalamus and the cortex and they are therefore the signal components least influenced by activity in the many other cortical areas that come later. These somatosensory responses can be easily identified in the signal space as prominent peaks by calculating and visualizing the so-called Somatosensory Evoked Potentials (SEPs) and Fields (SEFs), from the EEG and MEG data, respectively.

For the visualization of the SEPs and SEFs, we first calculate the average across the ST timecourses of all the EEG chanels and MEG channels, respectively. Then the average of the EEG (SEPs) and MEG channels (SEFs) is plotted separately for the time window 50 ms prior and 100 ms post-stimulus onset. In the same Figure, we also show the topographies of the averaged EEG and MEG data at the identified prominent peaks in the SEPs and SEFs. This is done for each subject and for each of the three groups of trials. Knowledge of the location of the thalamic and cortical generators and the physics of their electrical activity predict which components will be seen best with EEG and/or MEG. Previous studies align well with these expectations. Specifically, it is expected to find the (EEG) components P14, (P/N20), (N/P30) and (P/N40) in the SEPs (Politof et al., 2019). The labels P and N refer to the polarity of the peak (positive, negative) but note that for tangential superficial sources the P/N for EEG will appear in different electrodes. The number in the labeling of components corresponds to the peak latency. These cortical activations are expected to appear in the (MEG) components M20, M30, and M40 of the SEFs, i.e., the EEG and MEG are expected to “capture” the activity of the cortical generators that become active at 20, 30, and 40 ms (Kakigi, 1994; Papadelis et al., 2011; Politof et al., 2019). In contrast, no prominent M14 component (the SEF analog of the SEP P14) is expected because the location of the thalamus is close to the center of the head, which is bounded by the high resistivity and nearly spherical cranium; in such a geometry configuration the magnetic field outside the head, generated by electrical activity in the thalamus is almost zero, because the dominant direction of the electrical current dipole is in the silent radial direction (Kimura et al., 2008; Papadelis et al., 2012; Politof et al., 2021). In this study, we focus on the first early components at around 14 ms (P14) and 20 ms (P20, M20) since these are considered to be the first entries in the thalamus and the cortex, respectively.

### 2.5 Virtual sensors

#### 2.5.1 Linear spatial filters applied to a composite model signal

We have adopted a data-driven definition of separate linear combinations of EEG and MEG channels to extract from the raw EEG and MEG signals the early timecourses of the brain activity at the level of the thalamus and Somatosensory cortex. The linear combinations of channels are defined at two times. First around 14–15 ms when the evoked activity reaches the VPL nucleus of the thalamus. The VPL is the end-point of the spinothalamic tract: it is the main gate for activity elicited by somatosensory stimuli to enter the thalamo-cortical circuit. Within a few ms, around 18–20 ms the evoked activity reaches the primary somatosensory cortex, Broadman Area 3b (BA3b). We will simply refer to the output of these two linear filters as the activity of the thalamus and cortex hereafter, providing further clarification wherever this is needed. We will first illustrate the logic behind the approach we have taken and justify the label as virtual sensor (VS) of our seemingly simplistic linear spatial filter we have designed. The approach is illustrated using a simple, yet realistic scenario, which approximates reasonably well the findings of many earlier studies of the variability of responses to somatosensory stimuli (Ioannides et al., 2002; Stephani et al., 2020). We know that the EW stimulation elicits the first evoked responses at the level of the thalamus at the VPL around 14–15 ms and few ms later the evoked response can be identified at the cortical level in the Broadman Area (3b). Each of these responses is a focal well-circumscribed activation that can be approximated well by a single equivalent current dipole (ECD).

We generate a model signal, for the system we wish to study, which we will refer to hereafter as the composite model signal (CMS) to contrast it with the actual measurements which we will refer to as the measured response to EW (MRtEW). The CMS is a mixture of a computer-generated signal (sum of two ECDs, one for the thalamus and the other from the cortex) to which we add realistic background brain activity from a segment of the pre-stimulus period of the MRtEW. The CMS for each ST is the sum of two computer generated signals and a segment of pre-stimulus period of that trial (from −70 to −20 ms with respect to the onset of EW stimulus at 0 ms), from the actual background of the preprocessed EEG and MEG data. Each ECD of the CMS is modulated by a Gaussian function with fixed shape, full width of 5 ms at 5% of the maximum and strength adjusted so that the signal generated at the peak is comparable to the signal at the peaks of the MEG and EEG signal of single trials. A jitter is allowed in the latency of each ECD onset, by varying the latency of the center of the Gaussian form factor within a range of 4 ms, i.e., 2 ms on either side of the mean latency of 15 ms for the thalamic ECD and 20 ms for the cortical ECD. The random latency within the 4 ms range is varied independently for the thalamic and cortical ECDs. The location and direction of the ECD are fixed to what others have reported before for these two centers from EEG (Porcaro et al., 2008; Götz et al., 2014), MEG (Tesche, 1996) and MEG with fMRI (Papadelis et al., 2012) studies. The forward problem computation employed a head model constructed using a realistic three-layer (brain, skull, scalp) Boundary Element Method (BEM) volume conduction model of the head, based on a template MRI fitted to the electrode montage used for subject 1. The EEG and MEG signals were computed at the same locations as were given for the MRtEW experiment: 71 electrodes on the scalp for the EEG and 271 locations of the radial gradiometers inside the dewar, for the MEG sensors. The EEG- and MEG- CMS signals for one random ST are presented in the plots A and C of the Supplementary Figure S4, respectively. Plots C and D of the Supplementary Figure S4, show EEG and MEG topographies, respectively, for different time points at and around the peak latencies of the input ECD signals, illustrating the emerging distinct patterns (as expected from physics) at the ECD's peak latencies for that ST (e.g., 15 and 20 ms)

**Figure 2:**
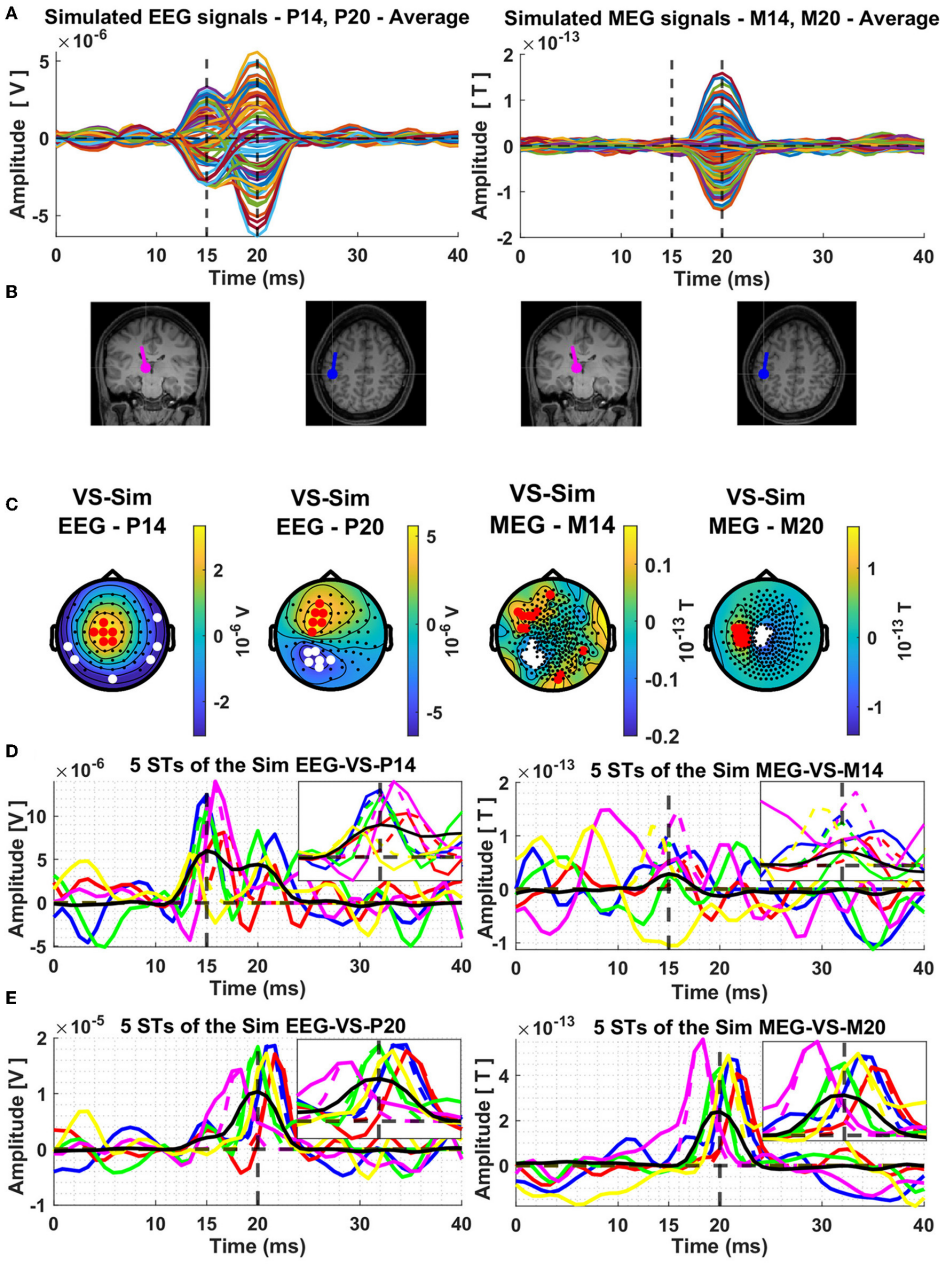
Average composite model signals (CMS) and source reconstruction of the modeled thalamic and cortical equivalent current dipoles (ECD) using the Virtual Sensor (VS) method. The average CMS signals across the 239 trials are shown in Panel **(A)**, for EEG and MEG, in left and right columns, respectively. Panel **(B)** depicts the location and orientation of the ECDs plotted on the template coronal MR image for the thalamic dipole and axial MR image for the cortical dipole. Constructed Virtual Sensors are shown in Panel **(C)**, with red colors indicating the selected sensors with positive and white color the sensors with negative amplitude. The VS extracted activations of the ECD in the thalamus [Panel **(D)**] and in the cortex [Panel **(E)**], using the EEG (left column) and MEG (right column) generated data are shown for five random Single Trials. In all four plots shown in Panel **(D, E)**, the same five colors are used to differentiate between the VS signals of each ST, black solid lines show the average of all the 239 trials and the dashed vertical lines indicate the mean time of the peak activity of the input ECD signals across all the trials. The box in the top right quadrant of each plot of Panels **(D, E)** features zoom versions in the period 10–20 ms and 15–25 ms for the thalamic and cortical VS, respectively.

The next display, Figure 2, shows results for the average signal. The resulting butterfly plots for the average signals are displayed in row A of Figure 2, on the left half for the EEG and on the right half for the MEG. The dipole locations are shown in the next row (B) of the Figure 2 and in the third row (C) the topographies at the peaks of the thalamic and cortical ECD activations, which correspond exactly with the extrema of the butterfly plots shown in row A of Figure 2.

In left half of Figure 2B, the two displays show the thalamic ECD in a coronal MRI slice (at the level of VPL) and the cortical ECD in a fairly superficial axial MRI at the level of BA3b; The anatomy in Figure 2B is placed under the EEG butterfly plot (Figure 2A). Each EEG topography (Figure 2C) is placed directly below the ECD responsible for the corresponding peak latency. In the right half of Figure 2B, the display of the two ECDs is repeated again for the corresponding MEG butterfly (right half of Figure 2A) and topographies (right half of Figure 2C).

The MEG activity generated by the nearly radial thalamic ECD is much weaker, with a ratio of 10:1, compared to that generated by the nearly tangential cortical ECD. As a result, only a slight swelling but no clear peak is seen at the latencies of peak thalamic activity. A clear and nearly symmetric dipolar pattern is seen in the topography of the MEG signal at the latency of the peak of the cortical dipole, which is rotated by 90 degrees relative to the pattern of the EEG at the same latency. These patterns are as expected from physics, and they can be used to prescribe a completely data-driven and robust sensor-based analysis suitable for both average but also for ST signals. The procedure for designing such a robust spatial filter, will be described after we use the knowledge of the laws of physics to justify labeling these spatial filters “virtual sensors” for the underlying active sources.

#### 2.5.2 Virtual sensor interpretation of our spatial filters

In EEG and MEG we are usually concerned with frequencies below 1 kHz, a range where active changes in the current density distribution in the brain are slow enough for the time derivatives to have negligible effect, i.e., for the quasistatic approximation of Maxwell's equations to be valid (Hämäläinen et al., 1993). In the quasistatic regime we can consider topographies of sets of MEG, EEG or even intra cortical EEG (icEEG) or intra cortical MEG (icMEG) as (nearly) equivalent representations of the current density changes that took place at that instant). Without any loss of generality we can assume that for each single trial we have the timecourses $y_{j}^{1}\left(t\right)$ and $y_{j}^{2}\left(t\right)$ for each of the two generators, respectively, that are scaling some normalized topographies, ${\hat{Y}}^{1}$ and ${\hat{Y}}^{2}$, representing respectively the imprint of the sub-cortical activity starting in the brainstem and culminating in the thalamic peak at 15 ms (${\hat{Y}}^{1}$) and the cortical activity (${\hat{Y}}^{2}$) starting around 18 ms in BA3b and peaking at 20 ms. Under this scenario, both the real (MRtEW) and composite (CMS) signals, can be written as *S*_*jl*_ (*j* = 1 to *N* trials and *l* = 1 to *L* sensors),


(1) $$\begin{array}{l} S_{jl}\left(t\right)=y_{j}^{1}\left(t\right){\hat{Y}}_{l}^{1}+y_{j}^{2}\left(t\right){\hat{Y}}_{l}^{2}+\Theta_{jl}\left(t\right), \end{array}$$

Equation 1 is an “exact” representation of the CMS and MRtEW signals, in a trivial sense: we define it to be so, by setting the remainder Θ_*jl*_(*t*), to represent the effect of all other sources and any instrumental or environmental noise that are present at time *t*.

Note also that the formulation can be expanded to many more sources, up to the total number of sensors. A vast range of standard linear algebra techniques can then be used to extract from the data a wide range of equivalent representations of the measurements. The mathematical analysis can be considered as a decomposition of the signal, for example as a spectral decomposition with components ranked according to their strength (amplitude of the eigenvalues) of the corresponding linear system. From the point of view of physics, the raw signal is the result of applying mathematical operator based on the laws of electromagnetism to the underlying generators, which involve the details of the conductivity profile of all head compartments between the active generators and the sensors. For an excellent concise summary of how the basic ingredients of our measurements, the electric and magnetic field relate through integral representation of the generators see the classic paper of Sarvas (1987).

Measurements for each one of the four modalities (EEG, icEEG, MEG, and icMEG) relate to integrals of functionals over the space where primary currents can exists. These functionals depend inversely on the distance from the source and either the divergence of the primary current (or equivalently the Laplacian of the electrical potential) in the case of electrical measurements (EEG and icEEG) or the Curl ∇_*x*_ of primary current in the case of magnetic measurements. More specifically for the non-invasive EEG/MEG signals, the Surface Laplacian (SL) and *V*_3_, respectively. The SL is the radial contribution of the divergence of the primary current density vector on the scalp ($\frac{\partial }{\partial r}\left(\hat{r}.{\vec{J}}^{p}\left(r,t\right)\right)$) (Hjorth, 1975; Kayser and Tenke, 2015). *V*_3_ is the radial component of the Curl (at the measurement point) of the Curl of the functional of the primary current density at the limit as we approach the source, which for a focal superficial generator aligns with the projection of ${\vec{J}}^{p}\left(r,t\right)$ on the tangent plane (Hosaka and Cohen, 1976; Ioannides, 1987; Haberkorn et al., 2006).

#### 2.5.3 Virtual sensor construction

The timing of the VS was fixed from the average signal and the topography was plotted time slice by time slice. Two periods of stable topographies were identified for the EEG and MEG signal, one around 15 ms and the other around 20 ms. The period of stability varied for each case; it was longest and the pattern most distinctive for the EEG signal at 15 ms and for the MEG signal at 20 ms. The candidate members of each set can be seen clearly in the topographies extracted from the average signals (see Figure 2C). For the EEG, the topography around 15 ms is particularly well-defined, hence revealing the candidate channels for the EEG thalamic VS. For the MEG, the topography is particularly well-defined around 20 ms, hence revealing the candidate channels for the MEG cortical VS. The EEG topography at 20 ms is reasonably well defined too, but not as clear as that of MEG. After this initial inspection, the members of each of the two sets were selected automatically on the basis of the consistency of the activity of each sensor in the critical latency periods for each VS. The selection was based on their signal power (SP), noise power (NP), and their ratio, signal to noise ratio (SNR), computed across the trials and at all time points of the trial (−0.1 to 0.2 ms) using a moving window of three samples (2.5 ms). The methods for the computation of the SP, NP and SNR has been described before (Liu et al., 2003). In panel C of the Figure 2 the selected sensors are shown for each of the four topographies by the red (positive) and white (negative) filled circles, superimposed onto the topographies at the time of the average peak signals. Each VS, for each topography or Type T, was constructed as the difference of the mean values of two linear combinations of signals of opposite polarity, a set of *N*_*A*_ positive values and a set of *N*_*B*_ negative values.


(2) $$T_{{\text{x}}_{vs}}(t)=\frac{1}{N_{A}}\sum_{i=1}^{N_{A}}{\;}_{A}^{T}{\text{X}}_{i}(t)-\frac{1}{N_{B}}\sum_{j=1}^{N_{B}}{\;}_{B}^{T}{\text{X}}_{i}(t),$$

The terms ${\;}_{A}^{T}{\text{X}}_{i}\left(t\right)$ and ${\;}_{B}^{T}{\text{X}}_{j}\left(t\right)$ are used to represent the time domain signals of each channel and in each group, respectively, with *t* used to denote the time point in the time period of the trial (−0.1 and 0.2 s before and after the stimulus onset). The superscript *T* refers to the virtual sensor type (EEG or MEG and latency), the subscripts *A* and *B* are used to show the two groups of channels (positive and negative amplitude channels, respectively) as selected by the definition of the VS, while the index *i* denotes the number of the channel in the group. The terms *N*_*A*_ and *N*_*B*_ is the total number of channels in the groups of channels *A* and *B*, respectively. Finally, the term *x*_*vs*_(*t*) at the left-hand side of the equation is the computed VS output. Once the virtual sensor is defined it can be applied to the signal across all latencies of either the average signal, selected averages (e.g., defined by different tasks or stimuli) or for STs.

The timecourses of the thalamic and cortical generators for the five random single trials and the average of the 239 STs are estimated by applying each of the four types of VS to the corresponding signals. The results are displayed in the last two rows Figure 2. Figure 2D shows the estimates for the thalamic response extracted from the VS defined for the signal at 15 ms, for EEG on the left and MEG on the right. Figure 2E shows the estimates for the cortical response extracted from the VS defined for the signal at 20 ms, for EEG on the left and MEG on the right. For ease of comparisons between modalities (EEG/MEG) and studying the interference of contributions from each generator to the VS of the other generator, the same five random STs are used in each display, each ST keeping the same color in all displays, with the corresponding VS extracted from the average signal shown in black. Also, for each ST the Gaussian is normalized to the maximum value of the ST in the display window and printed in the same color as the ST VS signal providing a visualization of the distortion of the shape of the VS output relative to the shape of the form-factor of the ECD. Finally, the period where the ECD is active is enlarged in the insert of each plot, for the thalamic VS (Figure 2D) from 10 to 20 ms and for the cortical VS (Figure 2E) from 15 and 25 ms, for a clearer view of the input Gaussian-Shape ECD signal and the corresponding VS output.

The inspection of the results for STs in Figures 2D, E show an excellent performance as a detector of the ECD evoked responses, when the response is comparable or higher than the background interference, this typically occurs when the ECD is at about half its strength or higher. This is the case for the thalamic activity using the EEG VS at 15 ms and for the cortical activity using the MEG VS at 20 ms. We note that the jitter in latency lowers the peak activity of the average and makes the shape wider. There is also some evidence that there is some leakage in the thalamic VS from activity of the cortical dipole, while for the MEG VS the interference from the thalamic ECD activity is smaller. There is also a response in the VS output from the background activity, which could be partially generated by noise in the background signal, but also by activity in BA3b and nearby areas that are known to be active in the pre-stimulus interval (from where the background is taken).

This simple example demonstrated how the VS was applied to the CMS data. The same procedure for defining the VS, was applied to the real data for the set of three subjects and the selected groups of trials (three groups for each subject, see Supplementary Table S1) without movement artifacts as described in Section 2.3. Starting with the identification of the VS from the average across the trials in each group and then applying the VS to each ST in that group of trials to estimate the ST generator's activity. There are some differences in the real system, for example the presence of other activations in the brainstem preceding the thalamic one and activity in other nearby cortical areas to BA3b which influence the latency over which the VS is stable, as will be explained in the results Section 3.2.

### 2.6 Clustering of STs

In order to deal with the spatiotemporal variability of ST responses, we perform clustering on the ST timecourses extracted by the VS. The grouping of the ST timecourses of the same regional response is a straightforward and relatively easy way to explore the ST variability (Laskaris et al., 2004; Zainea et al., 2005). Hence, we wish to group VS-trials with similar responses together. In order to accomplish that, we utilize Graph Theory (Bondy and Murty, 1976; West, 2001; Mieghem, 2010) to construct a network that captures the pairwise similarity of the data and use well-established graph clustering algorithms to do the grouping. In this network, the VS-trials constitute the nodes, while links quantify the similarity between them.

We aim for the network that we construct to capture similarity on specific temporal windows, centered around specific responses, hence we refrain from using the whole trial as input. We use the a priori knowledge of when the peak of the response of interest is expected. In order to focus on the specific response (e.g., P14, P20) we multiply the signal with a Gaussian function centered at the expected latency of the response's peak and with standard deviation σ = 10 ms. Thus, we retain 95% of the signal in a 2σ = 40 ms window around the response (20 ms prior and 20 ms post the peak, see Supplementary Figure S6). In this way sharp edges are avoided (e.g., using a 40 ms rectangular window) and also emphasizing similarities close to the center of the window while de-emphasizing in a smooth way the tails as we approach the edges of the window. Avoiding abrupt discontinuity ensures that transient large anomalies do not significantly affect the results.

In terms of which similarity function to (pairwise) compare our data with, we tested popular choices (e.g., Euclidean distance, Gaussian kernel, etc.) and settle for Pearson correlation coefficient, a linear and model free measure commonly used for quantifying statistical dependencies between brain signals in the time domain (Chiarion et al., 2023):


(3) $$\begin{array}{l} r_{xy}=\frac{\sum_{1}^{n}\left(x_{i}-\langle x\rangle \right)\left(y_{i}-\langle y\rangle \right)}{\sqrt{\sum_{1}^{n}{\left(x_{i}-\langle x\rangle \right)}^{2}\sum_{1}^{n}{\left(y_{i}-\langle y\rangle \right)}^{2}}}, \end{array}$$

where *n* is the length of the trials, and 〈.〉 indicates average over time. Since we are interested in identifying trials with similar responses, negative values of correlation are simply neglected.

After constructing the correlation matrix, which contains the similarities among VS-trials, we use one of the most common methods to sparsify the network. The *k*-Nearest Neighbor network retains the *k*-strongest connections for each node. That way, low-weight (low similarity) edges are neglected and the analysis becomes much clearer. With some exploration on our data, we chose *k* = 10, which guarantees that all the resulting networks are connected, while retaining as few edges as possible (Luxburg, 2004).

We use the Louvain algorithm (Blondel et al., 2008) to group similar trials together. This is the most popular algorithm that greedily maximizes modularity (Newman, 2006). Modularity (*Q*) is a measure of “modular” structure that utilizes “within cluster”-“between clusters” edges and ranges between [−1/2, 1]. The more modular a network is, the higher its modularity. The Louvain method initially treats each node as a community and iteratively groups communities together, as long as an increase in modularity can be obtained. In order to overcome the arbitrariness that Louvain has, which arises from the random choice of the algorithm initialization, we appeal to statistics. We execute a large number of Louvain runs (300 runs) and construct an allegiance matrix, showcasing the number of times that trials *i, j* were in the same community. Finally, we employ the Louvain one last time on the allegiance matrix. The clustering results are visualized in the form of a Consensus matrix (Rasero et al., 2017). Clustering analysis is performed independently on the ST signals extracted by the three VS as described in Section 2.5 as constructed for each of the three group of trials in each subject.

### 2.7 Connectivity estimation

In order to estimate the functional connectivity between the thalamus and the somatosensory cortex, we used the best VS for each case. For the thalamus, the only available choice is the VS derived from the P14 peak of the EEG data. For the cortical (S1) estimate we have two available choices the P20 and the M20 peaks respectively. We selected the M20 peak because of the highest sensitivity of MEG for superficial sources which allows it to better separate contributions from simultaneous sources.

For the computation of the connectivity we employed two different connectivity measures. The first measure, Pearson's correlation coefficient (CC), is a linear measure of similarity between two vectors (see Equation 3). The second method, mutual information (MI), is a non-linear connectivity metric from Information Theory (see Equation 4).

MI is a generalization of the Pearson's correlation between two random-variables *x*, and *y*. The MI is a model-free connectivity metric since it does not assume any law for the marginal and joint distributions of *x* and *y* (Bastos and Schoffelen, 2016). A consequence of being model-free is that both linear and non-linear relations are accounted by MI. The drawback of this metric is that estimating the underlying probability distributions and joint probabilities require a large amount of data, otherwise, the MI estimations will suffer from high bias. Many estimators of MI were proposed and usually, they demand a high computational power (Moon et al., 1995; Kraskov et al., 2004).

To overcome the computational cost of estimating MI, a recent estimator (Ince et al., 2017) uses the fact that the MI can be estimated analytically for data that follows a normal distribution. In the latter case, the mutual information will only depend on the covariance matrix (Σ) between *x* and *y* and it is given by Equation 4.


(4) $$\begin{array}{l} \text{MI}\left(x,y\right)=\frac{1}{2}\left[{\left(2\pi e\right)}^{2}|\Sigma |\right], \end{array}$$

This estimator makes use of functions that projects the probability distributions of the data into a space where they follow a normal law. Those functions are called *Gauss-Copula* functions (hence the Gaussian-Copula estimator). In the present work, we made use of this estimator in order to compute the mutual information between power/coherence and stimulus. From now on, we refer to it as Gaussian Copula Mutual information (GCMI). This approach has been already adopted in recent works in neuroscience (Zbili and Rama, 2021; Ashrafi and Soltanian-Zadeh, 2022; Combrisson et al., 2022).

Both measures, CC and GCMI are employed to quantify the time-delayed similarity between the ST signals extracted using the VS for the P14 and M20 components, which represent the activity of the thalamus and S1 cortex, respectively. Connectivity is estimated for each ST with a moving window of 12 ms and a step of 0.8 ms, while introducing time delays ranging from −20 to 20 ms in increments of 0.8 ms. In the estimation of the time-delayed CC (tdCC) and time-delayed GCMI (tdGCMI), the estimated signal of the thalamus (VS-EEG-P14) serves as the reference signal, while the estimated signal of S1 cortex (VS-MEG-M20) as the recipient signal. Positive time delays in the estimated connectivity values, indicate a temporal sequence where the thalamus is activated before the cortex, establishing an influence directed from the thalamus to the cortex. Conversely, negative time delays indicate that the cortex exerts an influence on the thalamus.

For each of the three groups of trials and for each of the three subjects, the time-delayed tdGCMI and tdCC were estimated for all the STs. Then the cluster of trials in a group, representing better the activity of the thalamus was selected. The clustering membership of the VS-P14 ST signals is used for the analysis. The criterion used for the selection of the cluster was that the positive peak was around the expected timing of the first early response in the thalamus around 14 ms (see Supplementary Figure S5). The connectivity estimates were averaged across the trials belonging to the selected cluster. Finally, statistical analysis was performed between the averaged connectivity values to identify the regions (time-latency and time-delays) with significant (*p* < 0.0005) tdGCMI and tdCC values.

### 2.8 A novel connectivity analysis

To assess the nature of connectivity between the subcortical and cortical regions, we applied the following additional analysis steps. First, we calculated the tdGCMI and tdCC 2D-connectivity maps. Displaying the scatter plot of these values, i.e., showing each single trial as a point in a 2-dimensional plot with the horizontal coordinate representing the tdCC value and the vertical component the tdGCMI values provides a measure of symmetry/asymmetry in the positive and negative tdCC values. As a first approximation positive and negative tdCC values can be interpreted as evidence for excitatory and inhibitory influence between the two areas, respectively. No such interpretation is available for the non-linear tdGCMI values. The value of the GCMI does not depend on any time ordering within the time series, but only to the distribution of their values. As a consequence the result has a probabilistic interpretation and hence only positive values. By combining the two measures we can get linear and non-linear effects and at least a hint of the balance between excitation/inhibition in the connectivity of the two time series. We combine the two measures by multiplying the GCMI values with the sign of the CC values for each ST. Assuming that the excitatory/inhibitory influence of the tdCC can be generalized in the non-linear tdGCMI, the product of the two provides an estimate of the excitatory/inhibitory of the more generalized non-linear tdGCMI. A color coded display of the resulting MI map shows the latency delay relationship and hence the direction while the color denotes both the strength and the nature (excitatory or inhibitory) of the influence of one area to the other. We will demonstrate in the Results section examples of this novel use of linear and non-linear aspects of connectivity analysis.

## 3 Results

### 3.1 Somatosensory evoked potentials/fields

**Figure 3:**
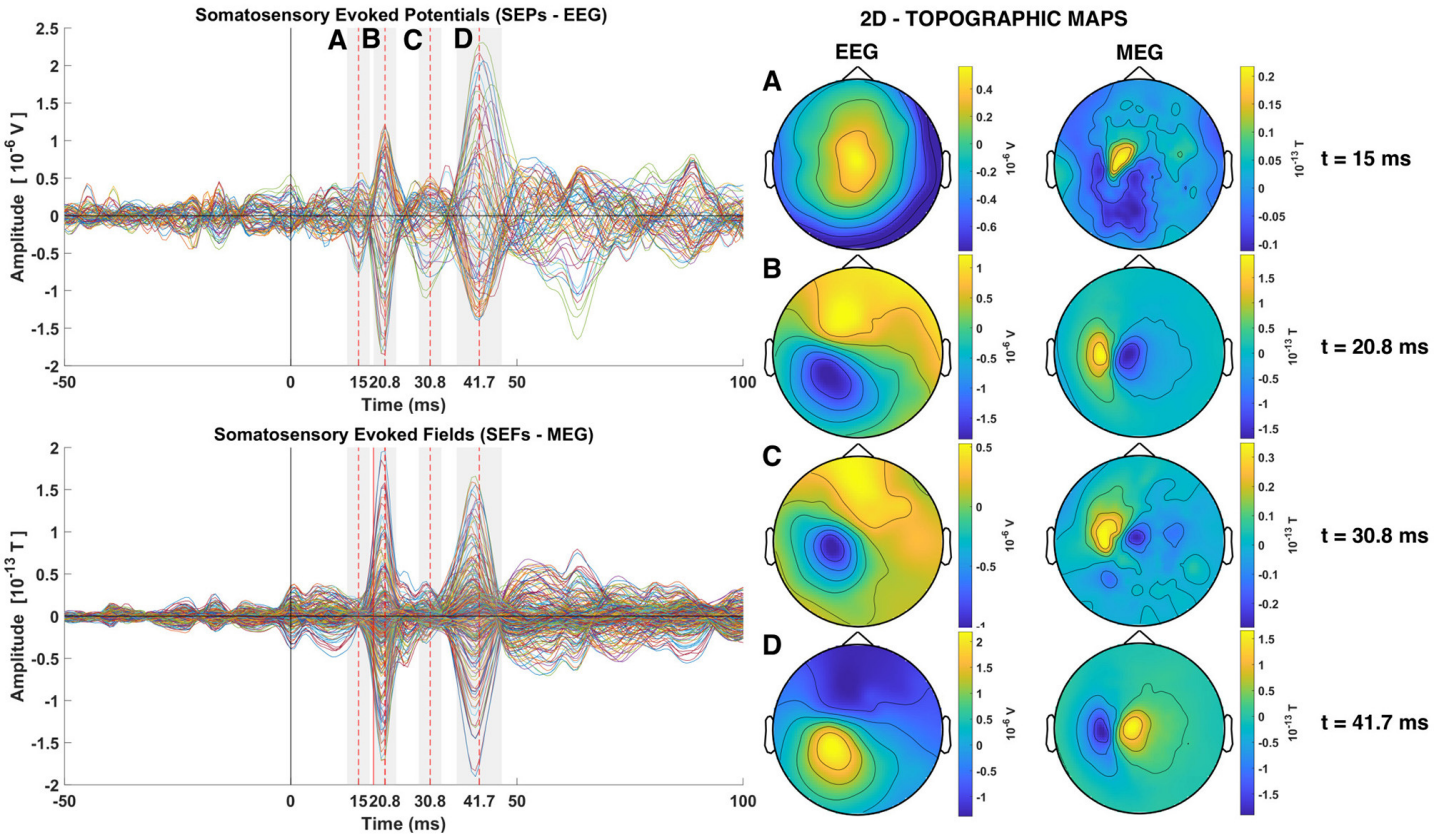
Somatosensory Evoked Potentials (SEPs) and Somatosensory Evoked Fields (SEFs) averaged from the group of trials 1 of subject 1 **(left)**. The two rows of the left column shows the timecourses of the average across all trials for all channels superimposed (Butterfly plots), with EEG on the top and MEG on the second row. Dashed red lines show the times of the P14, P20, P30, and N40 components. The topography at the latency of each one of these four peaks is shown in the **Right** column of the Figure, with the EEG in the penultimate and MEG in the last column.

Somatosensory stimulation gives rise to the so called somatosensory evoked potentials (SEP) and somatosensory evoked fields (SEF) recorded by the EEG and MEG sensors, respectively. Even with the strong EW stimuli used on our data it is difficult to identify the SEF and the SEP responses in individual STs, although possible to do so, at least for the SEF if one knows in advance where to look. If one averages a number of STs the SEFs and SEPs are enhanced and they appear as strong peaks in the first 60–80 ms, well above the background activity before and after this period (Zainea et al., 2005). Human Cortical SEPs are separated into short latency potentials (occurring in the range of 0–40 ms post stimulus onset) and long latency potentials (occurring after 40 ms) (Allison et al., 1991). Figure 3 shows timecourses and topographies for the average of one of the three groups of trials of subject 1 with 239 STs (see Supplementary Table S1). The SEPs and SEFs are clearly seen in the butterfly plot on the left part of Figure 3, marked by the shadowed areas and their peaks, measured relative to the stimulus onset, with dashed red vertical lines. As expected, the first evoked response is only seen in the EEG with peak at 15 ms (marked with A in Figure 3), while all other peaks (B, C, and D at 20, 30, and 40 ms) are seen in both EEG and MEG. The right side of the Figure, shows the EEG and MEG topography of the signals at the times of the indicated peaks at the points A, B, C, and D.

The first short latency component in the SEPs is expected to occur at around 14 ms, namely the P14 component (Hanajima, 2004; Porcaro et al., 2008; Götz et al., 2014; Politof et al., 2021). In the SEPs shown in Figure 3 the peak of the P14 component is at 15 ms. The P14 component has been correlated to neural activity localized in the thalamus both from EEG and MEG studies (Hanajima, 2004; Kimura et al., 2008; Papadelis et al., 2012; Porcaro et al., 2013; Politof et al., 2019, 2021). No strong magnetic analog of the P14 component at 15 ms peak is visible in the SEFs (MEG data), except for an intriguing well circumscribed feature with nearly an order of magnitude weaker activity than the next peak a few ms later, at 20 ms. The next expected short latency potential, namely the P20 component and M20 component, shown in the SEPs and SEFs respectively, occurs at 20.8 ms. This peak activity has been localized in area S1 (Gaetz and Cheyne, 2003; Papadelis et al., 2011; Porcaro et al., 2013; Hari and Puce, 2017; Antonakakis et al., 2019; Politof et al., 2019). More specifically, the P20 and M20 have been localized independently in the Broadman area 3b, the primary somatosensory cortex (S1) (Allison et al., 1989; Forss et al., 1994; Kakigi, 1994; Ioannides et al., 2002; Gaetz and Cheyne, 2003; Antonakakis et al., 2019).

The topography of the P20 and M20 components exhibit a dipolar pattern. By comparison of the EEG and MEG topography at 20.8 ms, it can be seen that the two dipolar patterns created in the two modalities are orthogonal to each other, as expected. The focus of this study is on the P14 and P20/M20 components, which exhibit the expected pattern for a thalamic (nearly radial) generator for P14 and a mostly tangential S1 generator for P20/M20. At 15 ms the mainly radial component of thalamic activity is generating a widespread pole in the top of the head in the EEG distribution butterfly plot of the EEG. There is no discernible peak in the MEG, although a distorted dipolar pattern is seen in the MEG topography. The P20/M20 peaks are consistent with a tangential superficial source and its timing agrees with all the a priori knowledge about S1 activation. The later peaks, athough not at the focus of this work, have also features worth noting. Both peaks have mainly dipolar patterns that are easily discernible in the P30/M30 and N40/M40 butterfly plots of SEPs and SEFs in the left column and the EEG and MEG topoplots on the right of the Figure 3 at times 30.8 and 41.7 ms, respectively. The last component (N40/M40) resembles in all its features the P20/M20 but with opposite polarity; the similarity of corresponding contours of the EEG and MEG topoplots at 20 and 40 ms (given the opposite polarity) is striking, suggesting a reactivation of S1 (with opposite polarity). The intermediate peak (P30/M30) is of the same polarity and similar in both EEG and MEG with the corresponding features of P20/M20, but with noticeable differences in the contour shapes, suggesting a mixture of nearby superficial, mostly tangential components and possibly some contributions from deep sources for the EEG.

### 3.2 Extraction of virtual sensor ST signals

Analysis of evoked responses using VS has been employed for the identification of early sensory responses for the somatosensory, auditory and visual cortex (Laskaris and Ioannides, 2002; Laskaris et al., 2004; Ioannides, 2006). In this study, three different VS were defined for the estimation of the generators of the components P14, P20 and M20 from the EEG and MEG data, respectively. Following the procedure for defining the VS as described in Section 2.5 the time slice at the center of the period with stable topography was identified for each of the three VS. In the case of the VS for P20 and VS for M20, this time slice was found to be on the rise and exactly three time-slices before the maximum amplitude of the targeted peaks, specifically at 18.3 ms compared with the peak at 20.8 ms (see Figure 3, middle and right columns). This is as expected because this is the first entry into the cortical surface. At that time, there is no other cortical source to interfere with. At the timing of peak activity other cortical areas (BA2, BA1, Papadelis et al., 2011) are already activated either via direct input from the thalamus or indirectly from the area BA3b. The detailed description of connectivity between the thalamus and the areas BA3a, BA3b, BA2 and BA1 is still not completely resolved and it is likely to be task dependent; it clearly needs further investigation.

In contrast, in the case of defining the VS for the estimation of the generator of the P14 component, the time slice at the center of the period with stable topography is the same as the time-slice of the peak (15 ms, see Figure 3 left column). This is in line with what is known regarding the activation of the brainstem few milliseconds before the thalamic activation. Earlier activation from the brainstem is more ventral than the thalamus and therefore will produce a similar topography as that produced by the thalamus. This explains why the timing of the stable topography coincides with the timing of the peak: it is because the thalamic activity is strongest, while that from the brainstem is diminishing.

**Figure 4:**
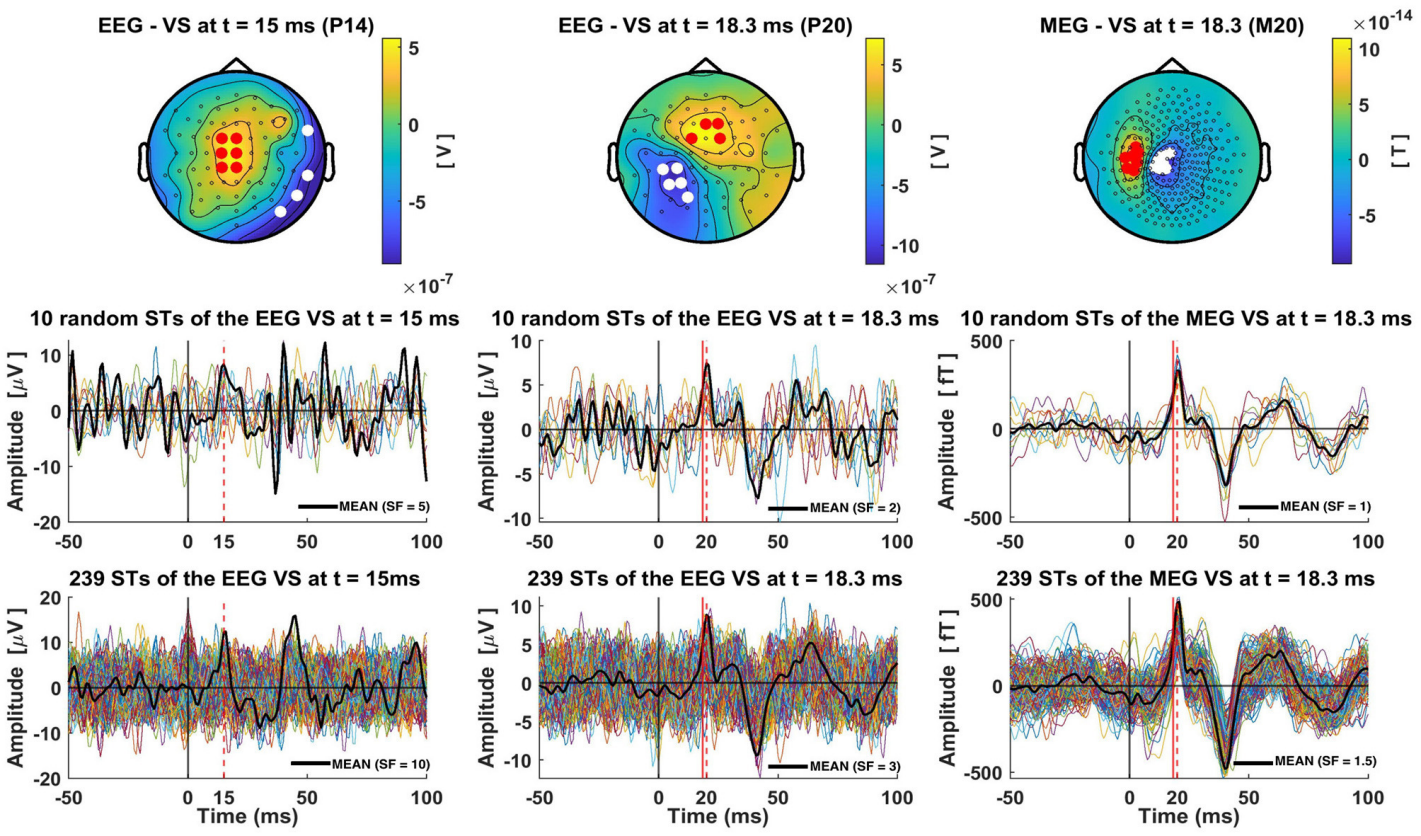
Single-trial estimations of the thalamic and cortical (S1) activity using Virtual Sensors (VS). The **top row** displays the three distinct VS constructions for capturing thalamic (EEG VS P14), cortical S1 activity from EEG data (EEG VS P20) and from MEG data (MEG VS M20), for subject 1 and group of trials 1 (239 trials). The times at which each VS is defined are indicated in the title of each topography. In these topographies, red and white dots are used to show the channels with positive and negative amplitude, respectively, that were selected for the construction of the generator-specific VS. **Bottom row** shows all 239 single-trial timecourses extracted by each VS, while the middle row presents ten random single trials. Red dashed lines show the timings at the peaks of P14, P20 and M20 components, and red solid lines mark the precise time-slice chosen for VS construction. In the case of P20 and M20 this red solid line is at 18.3 ms, while for the P14 no red line is used as the timing used for this VS coincides with that of the peak. A solid black line is used to show the average of the 10 **(middle row)** or 239 **(bottom row)** STs. These averages are scaled by a Scale-Factor (SF), indicated in the bottom right corner of each plot, to align the amplitude of the peak of interest with the peak amplitudes of the single trials, thereby highlighting the differences between single-trial and average peak amplitudes.

The average of the ST timecourses (black solid lines) extracted by each VS exhibit a clear peak at the expected time (marked as a red dashed line in Figure 4). Notably, the average has smaller peak amplitudes compared to the ST signals. This is evident by comparing the multiplication scale factor (SF) shown in the middle and bottom rows of Figure 4. For instance, in the case of the VS-EEG-P14 representing thalamic activation, SF was equal to 5 when averaging 10 trials, while it increased to 10 when averaging 239 trials. This is because there is some jitter in the timing of the peak at 15 ms. For the other two VS estimating the cortical activity at S1, the difference between SFs (middle vs bottom row) is not large, this is because the peak of the cortical activity at 18.3 ms is more consistent in terms of timing.

### 3.3 Results of clustering analysis

**Figure 5:**
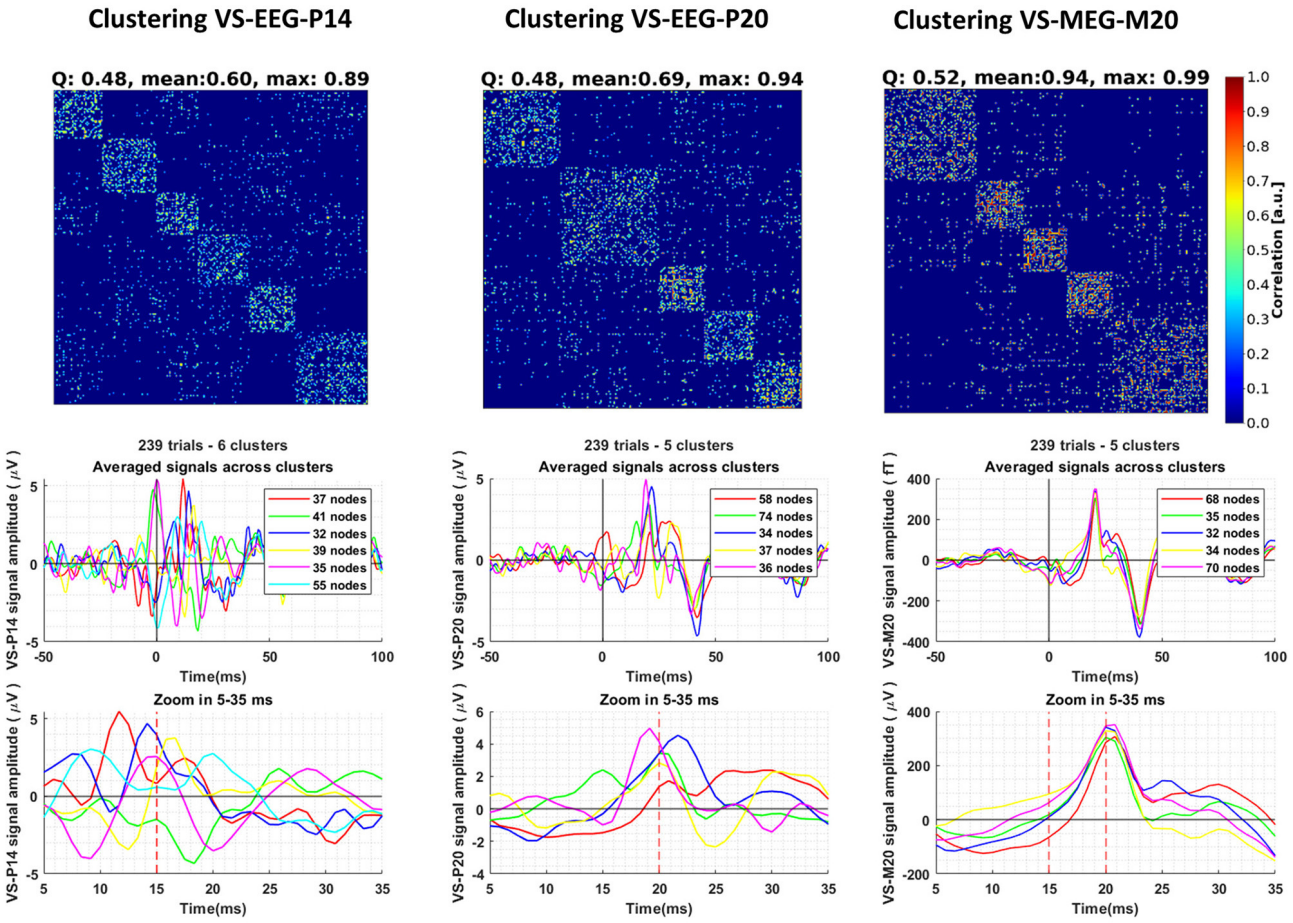
Clustering of the single-trial (ST) timecourses extracted using three different Virtual Sensors (VS) for Group 1 of Subject 1. The columns, from left to right, display the clustering results for the ST signals estimated by VS-EEG-P14, VS-EEG-P20, and VS-MEG-M20, respectively. The **top row** shows the Consensus matrix used to represent the clustering. The modularity (*Q*), mean and maximum similarity values for each clustering are shown in the title of the consensus matrix. In the **middle row**, the average ST signals for each cluster are depicted. The **bottom row** provides a zoomed-in version of the plots in the middle row between five (5) and thirty-five (35) milliseconds, emphasizing the differences in the averages among the different clusters.

Following the methodology described in Section 2.6, we conducted clustering analysis on the ST signals extracted using the VS. The clustering results, along with the corresponding averages of the ST signals within each cluster, are depicted in Figure 5. Consensus matrices were employed to visualize the clustering outcomes (Jeub et al., 2018). These matrices are square matrices in which each cell represents the pairwise similarity value between two ST signals, calculated using Pearson's correlation coefficient as described in Section 2.6. To facilitate pattern identification and emphasize groupings of high values, the rows and columns of the consensus matrix were rearranged by placing the trials (rows of the matrix) belonging to the same cluster adjacent to each other. By examining the consensus matrices, patterns of agreement among the clustering assignments can be discerned. High values along the diagonal indicate consistent clustering, while off-diagonal values represent disagreements or uncertainties in the clustering. With the reordering of trials, distinct square blocks emerge along the diagonal, clearly indicating the separation of clusters. It is worth noting that the STs of VS-EEG-P20 and VS-MEG-M20 exhibit higher maximum similarity (correlation) values compared to the similarity between the STs of VS-EEG-P14 (max: 0.94, 0.99, and 0.89, respectively, see top row of Figure 5). This difference in the maximum similarity values is an indication that the estimated ST timecourses representing the activity of the S1 cortex are more similar compared to the ST estimations for the thalamus. This is also shown by comparing the average signal of the clusters of each VS. It can be observed that the clusters for the VS-EEG-P14 exhibit noticeable differences in timing, polarity and amplitude of the peak compared to the average signal of the clusters in the other two VS (see Figure 5 bottom row). For example, the green line for the cluster with 17% of the STs, i.e., the 41 STs out of the total of 239 STs is identified by the thalamic VS to have opposite polarity to that of the overall average or most of the other clusters.

### 3.4 Connectivity results

**Figure 6:**
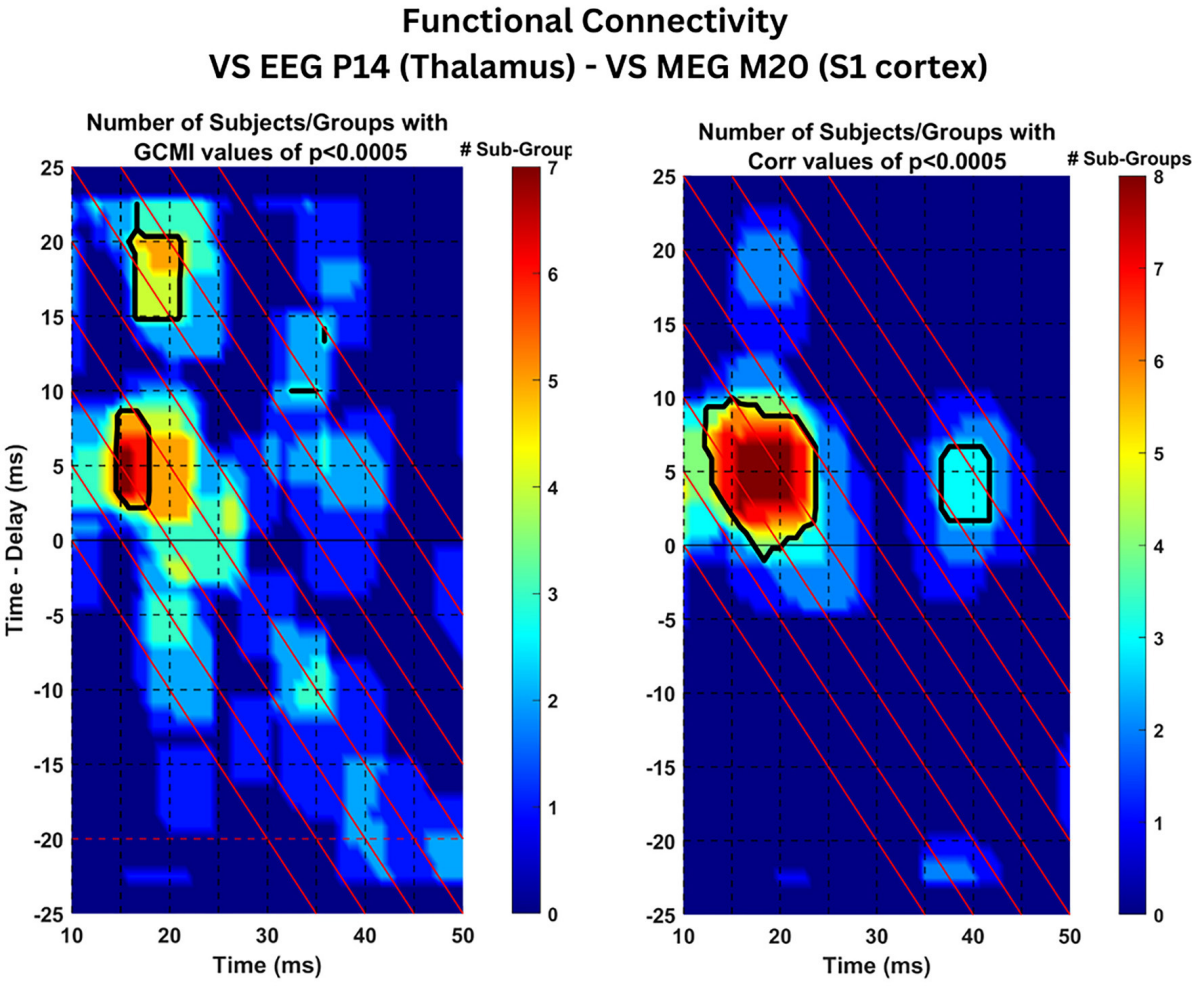
Connectivity results: Statistical analysis of connectivity values across subject. The Figure displays the estimated time-delayed Gaussian Copula Mutual Information **(left)** and Pearson's Correlation Coefficient **(right)** between the ST signals extracted by VS-EEG-P14 (representing activity in the thalamus) and the VS-M20-MEG ST signals (representing S1 cortical activity). The color coded values indicate the number of total groups of trials with statistical significant values (*p* < 0.0005) across the three subjects. The black contour lines show the regions with significant values in at least one group within each of the three subjects.

In the estimation of the functional connectivity, we use as reference signal the ST timecourse activity of the thalamic region as estimated by the VS-EEG-P14 while the recipient signal is the ST activity of the S1 cortex as estimated by the VS-MEG-M20. As explained in Section 2.8, for each subject and group of trials, the GCMI and CC connectivity is estimated for all the trials in the group. Then, for each subject and group the cluster representing the activity of the thalamus is selected (selected clusters are shown in Supplementary Figure S5). Then, statistical analysis is applied to the average of the 2D connectivity maps across the STs belonging to the selected cluster. Figure 6, at each point in time (*x*-axis) and time-delay (*y*-axis) shows the number of groups across all three subjects which have been found to have statistically significant connectivity values (*p* < 0.0005). Black outlines indicate the regions with statistically significant connectivity values in at least one group for every subject. Almost all three groups and all three subjects (7/9 for GCMI, 8/9 for CC) have shown significant connectivity values (*p* < 0.0005) at a latency (*x*-axis) of 15 ms (range 15–18 ms) and time delay (*y*-axis) of 5 ms (range 3–8 ms). This tells us that the activity observed at the thalamus at around 15 ms post-stimulus onset, is both statistically dependent and positively correlated with the S1 cortical activity present at 20 ms. We can infer that activity originating in the thalamus around 15 ms post-stimulus onset reaches the S1 cortex 5 ms later at ~20 ms post-stimulus onset. Next important result, only for the case of the GCMI estimator, around five out of nine groups across the three subjects show significant connectivity values at latency (*x*-axis) of 15 ms (range 16–21 ms) and time delay (*y*-axis) of 17 ms (range 15–20 ms).

### 3.5 Analysis of connectivity results

The results of the previous Section 3.4, demonstrated statistically significant connectivity values (*p* < 0.0005) across all the three subjects (seven out of the total nine groups of trials) at latency *t* = 15 ms and time delay τ = 5 ms (see Figure 6). Both metrics, tdGCMI and Pearson's tdCC resulted in high values and were statistically significant compared to other latencies and time delays. Hence, in this section we analyze the estimated tdGCMI and tdCC values around these times to further investigate the nature of the connectivity between the thalamus and S1 cortex by following the methodology as described in Section 2.8.

**Figure 7:**
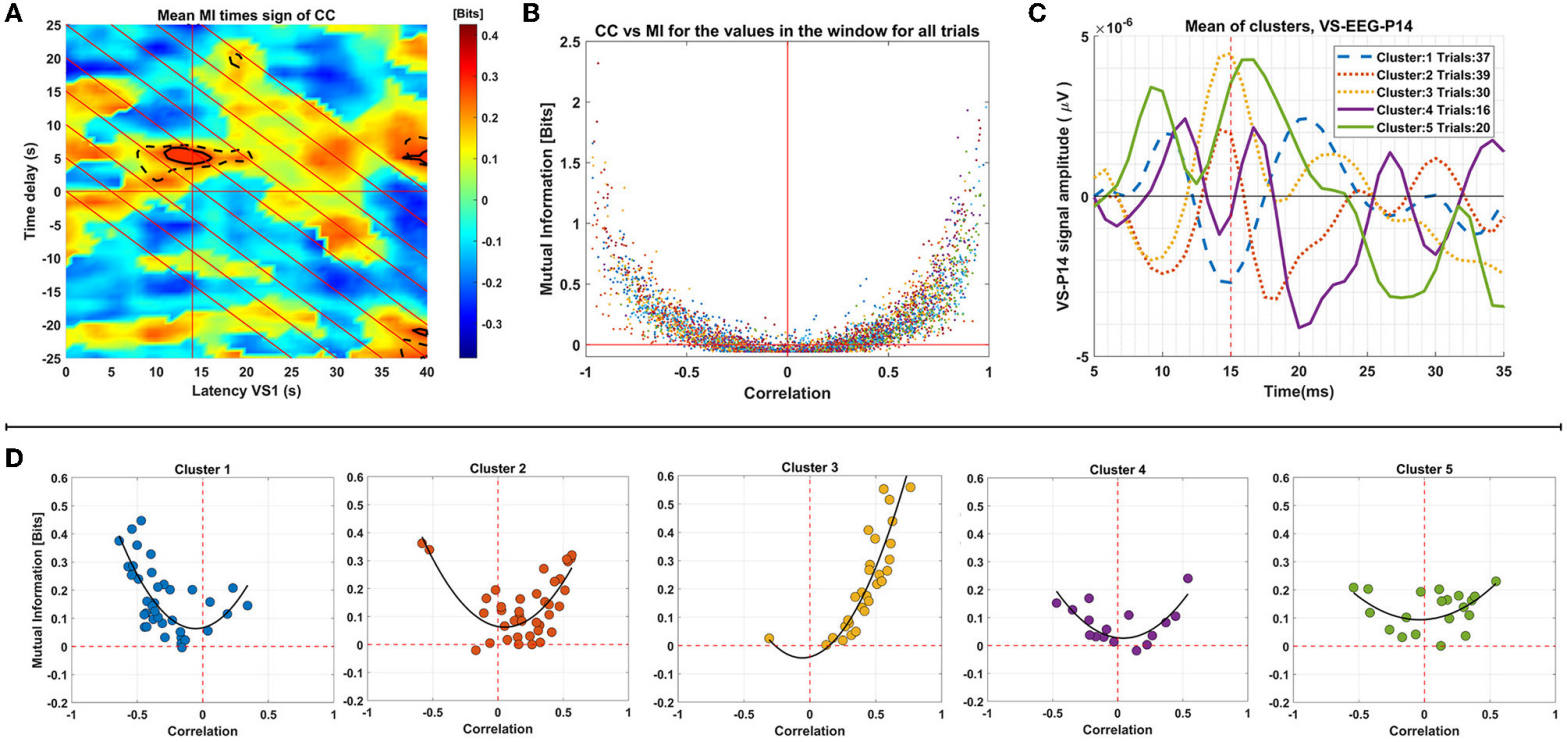
Analysis of connectivity results for group 2 of subject 1. This panel of plots presents the analysis of connectivity results for Group 2 of Subject 1. In Plot **(A)**, the MI values multiplied by the sign of respective correlation values are displayed. The region of significant MI values (*p* < 0.0005) is indicated by black solid outlines, while the region with respective significant correlation values is depicted as dashed lines. Plot **(B)** is a scatter plot demonstrating the relationship between the significant MI values enclosed by outlines (around 14 ms latency and 5 ms time delays) and the corresponding correlation values. Panel **(D)** shows the mean of the values for each trial, plotted in different graphs for each cluster. Plot **(C)** displays the mean of the single-trial VS-EEG-P14 values in each cluster.

Firstly, the tdGCMI values were multiplied with the tdCC values for each ST separately. Then we average these combined connectivity maps across STs of each cluster. In Plot A of Figure 7 we show these average results for the cluster 3, which was the selected cluster as described in Section 2.7 and used for the across subjects connectivity results presented in Section 3.4. The black solid and dashed outlines in Plot A show the statistically significant (*p* < 0.0005) tdGCMI values and tdCC values, respectively. The center of these outlined regions is at latency *t* = 15 ms and time delay τ = 5 ms as expected and based on the results of the previous Section 3.4. Having this information, we selected to further analyze the significant tdGCMI values within a square window of −2 ms on both directions and axes, centered at *t* = 15 ms (*x*-axis) and at time delay τ = 5 ms (*y*-axis). In plot B of Figure 7 the tdGCMI and tdCC values in the selected window and for all trials in the group (all clusters) were plotted as a scatter plot, with CC values on the *x*-axis and GCMI values on the *y*-axis. While tdGCMI is positive (by definition) the tdCC can also have negative values, ranging from −1 to 1. In addition, as depicted in plot B, the GCMI and CC values show a quadratic (U-shaped) relationship.

Subsequently, we computed the average values within the window for each ST, and these ST window averages were plotted in a similar manner, but in separate plots for the trials of each cluster (based on clustering of the VS-EEG-P14 ST signals as described in Section 2.6) as shown in Panel D of Figure 7. This analysis serves two purposes: firstly, it allows for visual inspection of the relationship between tdGCMI and tdCC values for all the trials together, and secondly, it enables comparison between trials of each cluster separately. By examining the tdGCMI-tdCC relationship in each cluster, potential differences regarding the nature of connectivity between the thalamus and the cortex can be revealed. It is obvious that for some clusters (clusters 2 & 3) there is a prominent lateralization of the correlation values on the right side (positive correlation) and in others (cluster 1) on the left side (negative correlation). For reference, the mean of the ST activation signals of the thalamus (VS-EEG-P14) for each cluster are plotted in plot C of Figure 7. It can be seen, that clusters with positive correlation values have the peak in the average of the STs at around 15 ms latency is positive, whereas for cluster 1, it is negative.

## 4 Discussion

In this study, a spatial filtering approach using VS combined with clustering was applied on simultaneous EEG and MEG recordings in response to EW stimulation, exciting electrically the median nerve. The results of the analyses led to five main findings. Firstly, it has been demonstrated that spatial filtering using VS can indeed be used to extract reliable estimates for the timecourses of known generators in the brain, superficial and deep. Secondly, constructing the activity of the thalamus and the cortex (S1), on a ST basis, reveals the existence of variability of the ST responses. Thirdly, clustering of the ST timecourses provides a principled classification of the variability of ST and reveals distinct processing sequences in each cluster. Fourthly, estimation of connectivity between the thalamus and the cortical area S1 reveals overall patterns (across the entire ensemble of STs), which is a composite of distinct patterns that can be disentangled within the identified clusters. The connectivity analysis across the entire array of STs identifies two distinct waves of thalamocortical activity both starting roughly around 15 ms (range 15–18 ms) for the first and (16–21 ms) for the second wave after the EW stimulus is delivered. The influence of these two thalamic activations arrive at the cortex (S1) with delays of 5 ms (range 3 ms to 8 ms) and 17 ms (range 15–20 ms) ms later. Finally, a more detailed analysis of the connectivity patterns is introduced, which combines the sign of the linear Pearson coefficient (tdCC) with the value of the non-linear tdGCMI results in their product. This combination allows, for the first time as far as we know, a novel discrimination of connectivity patterns, distinguishing excitation and inhibition from non-invasive recordings. Specifically, the novelty of the approach is being able to combine the linear and non-linear information captured by GCMI, with the directional aspect (sign) of the linear part of the relationship captured by CC which cannot be captured by GCMI. We construct from the average data the VS-EEG-P14 and VS-MEG-M20 so that the first entry to the thalamus (~15 ms) and the cortex (~20 ms), respectively, give rise to positive peaks. Using this convention, we find the thalamocortical interaction extracted from connectivity analysis on the average signal to be positive. The same analysis is repeated on a ST level, which can then characterize the nature of connectivity in each cluster, as will be discussed in Section 4.4.

### 4.1 Estimates of cortical and subcortical generators

Localization of the generators in the brain in response to an external stimulation and reconstruction of their timecourse activity using EEG and MEG data is a problem plagued by non-uniqueness, i.e., there is always more than one solution that can fit any given data exactly, even when accurate modeling of the head and knowledge of other parameters like conductivity of different compartments is available (Scherg, 1992; Mosher et al., 1999; Michel and Brunet, 2019; Asadzadeh et al., 2020). In this work, we rely on prior knowledge about the timing and location of the generators in the thalamus and the cortex and the expected topography each of this activity will generate, given the laws of electromagnetism. We focus on the latencies when the evoked response arrives for the first time at the targeted generators, because any feature in the signals generated by each generator is more likely to survive in the average across all STs, while that from other generators averaged out. Significantly this will happen even if the response is relatively weak in each trial or even if it occurs intermittently in some of the STs. Prior knowledge allows us to identify characteristic topographies at specific latencies of the average of the raw signal generated by activity of the targeted brain areas. The identified topographies in the average signal are used to define a spatial filter, which can extract this component even when it is buried in other much bigger signals. It is of course acknowledged that in each ST contamination from other generators will be present but in most STs it will be a small distortion of the timecourse around each activation of the targeted generator. In this sense, the process effectively removes the non-uniqueness aspect. No matter what the mechanism is, the same feature will be produced every time, possibly with some variation in strength and latency. These remaining variations are under the influence of the forward problem (computing the signal from the knowledge of the sources), which has a unique solution. In summary, the problem is transformed from a tomography problem to a detection of one or more features in the topography of the signal and the quantification of their strengths, but with some distortion from the influence of other sources which produce some contribution to the strength of the detected feature. In previous work we demonstrated that such approach works well for superficial sources using MEG data (Ioannides, 2006). In the current work, using simultaneous EEG and MEG recordings and a more automated way for the construction of the VS (see Section 2.5). We have taken a cautious approach to reduce noise and ensure that the VS were constructed for groups of STs with identical head position relative to the MEG sensor array. This was done by first identifying all periods of global disturbance in the MEG channels. These periods were then removed and VS were constructed using the average of the raw signal for STs between such disturbances. Several observations allow us to verify that the constructed VS capture accurately enough the ST timecourses of the generators (thalamus and S1 cortex). First and foremost, the estimated ST timecourses have peak activations at the expected timings. Secondly, the scalp topographies clearly depict distribution patterns of a deep nearly radially oriented source and a superficial source contralateral to the site of stimulation just above the S1 cortex, corresponding to the generator in the thalamic region and S1 cortex, respectively. In addition, the complementary information offered by the simultaneous EEG and MEG data further validate that the estimates indeed represent the activity of the thalamus and the cortex. For example, the P14 component which is seen in the SEPs at ~15 ms is not seen so clearly in the SEFs, suggesting that the generator of this component is somewhere near the center of the head and therefore an almost radially oriented source (Hämäläinen et al., 1993), leading to one more argument that this activity is originated from the thalamus. Also, the dipolar pattern seen both in the EEG and MEG scalp topographies at 20 ms have the same center location and they are oriented vertically to each other. This is a clear signature of a superficial strong generator with a strong tangential component. The later components (P30/M30 and N40/M40) are also are clearly visible as peaks in the average signal. These components were not discussed further in this manuscript except to state that their topographies are consistent with their reported localization in or close to the S1 area (Politof et al., 2019).

### 4.2 Thalamocortical connectivity from the entire ST ensemble

We next used the computed ST timecourses to calculate the functional connectivity between the thalamus and the S1 cortex in response to EW stimulation. Two connectivity metrics were employed, the simple linear Pearson's correlation coefficient and the non-linear measure Mutual Information. The results of both connectivity metrics revealed that at around 15 ms post-stimulus, activity of the thalamus travels to and reaches the cortical area S1 around 5 ms later. This result agrees with other past studies' results (Kimura et al., 2008; Papadelis et al., 2012; Götz et al., 2014; Politof et al., 2019). Götz et al. (2014), performed combined EEG/MEG source localization with single moving dipole model as well as fixed two dipole model (precortical and cortical dipoles). Their results showed dipole propagation from a precortical area near the thalamus to cortical regions with the precortical and cortical peaks reaching their maximum at 16 ms and 21 ms, respectively. The two fixed dipole models resulted in dipole clustering in the precortical and cortical regions. This dipole propagation from the thalamic region to S1 along thalamacortical fibers has been also shown in two other MEG studies in which the authors argue that this movement of the dipole corresponds to afferent information flow along the white matter thalamo-cortical fibers from thalamus to BA3b (Kimura et al., 2008; Papadelis et al., 2012). The consistency of the connectivity results across studies, including ours, further validates that the extracted ST activations using the proposed methodology, are accurate enough to be used for the study of connectivity between cortical and subcortical brain areas.

While all the previous methods simply identify a single thalamic excitation around 15 ms our connectivity analysis furnishes clear evidence for two distinct waves both starting around 15 ms. The first one starts a little earlier, around 14 ms and it is seen clearly in both the linear tdCC and the nonlinear tdGCMI estimates of connectivity. This first wave arrives to S1 at 20 ms and therefore is responsible for the P20/M20 peak. The second wave starts around 16 ms and it is seen clearly only in the nonlinear tdGCMI estimates of connectivity; there is only a faint indication for this second wave in the linear tdCC connectivity estimate. This is a clear indication that in the pair of early thalamic influences on S1 activity, a stronger non-linear component is present in the second compared to the first wave. To the best of our knowledge this is the first report from non-invasive electrophysiology of such dual early thalamocortical interaction. In this work we focused only on the well understood early thalamocortical interaction, leaving for a follow up study the detailed analysis of the second slower and non-linear interaction.

### 4.3 Variability of single-trial responses

A number of earlier studies have demonstrated the variability of ST brain responses to identical stimuli. Significant variability was demonstrated both in the spatial and temporal characteristics of ST responses to the same input stimulus (Liu et al., 1998; Laskaris and Ioannides, 2002; Goldman et al., 2009; Hu et al., 2011; Stephani et al., 2020; Waterstraat et al., 2021). Magnetic field tomography (MFT) (Ioannides et al., 1990, 1995) was employed in Ioannides et al. (2002) to reconstruct the cortical activity from ST MEG signals in response to EW stimulation. MFT relies on a non-linear algorithm to extract whole-brain tomographic estimates, independently for each timeslice of data. It can be applied to both the averaged and ST MEG signals and it has optimal properties for tomography (Taylor et al., 1999). Ioannides et al. (2002), focused on the activity in the primary (S1) and secondary somatosensory (S2) cortices. Firstly, comparisons of the waveforms of tomographic estimates in S1 for successive ST showed variability in temporal and spatial aspects. The tomographic solutions of the peaks with slightly different timings also suggest that there is some relationship between the timing of the peaks and the location of the generators in the brain. Futhermore, pattern analysis on the ST activation curves (ACV) in S1 and S2, allowed the extraction from the full ensemble of STs two distinct clusters. The first contained STs with clear peak at the expected latency of the first response to the stimulus in S1 (high power cluster). The second cluster corresponded to STs who showed little or no evoked response in S1 (low power cluster). Time-dependent Mutual Information (tdMI) analysis between the two cortical areas S1 and S2 was applied for the ensemble of STs belonging in each cluster separately. Interestingly, the connectivity results revealed two different communication modes between the cortical areas S1 and S2. The first communication mode corresponding to the STs in the high-power cluster is the canonical sequential activation from S1 to S2. In contrast, the second communication mode revealed by the connectivity estimates for the STs of the low-power cluster showed early and long lasting co-activation of S1 and S2. This finding of Ioannides et al. (2002) suggests that this variability between the ST responses cannot be random but is an intrinsic processing mechanism of the system. In the above 2002 study, there was no independent tdMI analysis for each ST and no estimation of thalamic ST activity, since no EEG data were available.

Although in this study we have not investigated the spatial aspect of the ST variability in terms of exact location of the ST generators, our results support the existence of variability among STs in terms of timing, polarity and duration of the peaks. More specifically, the constructed ST activations using the VS, show variability from trial to trial. This variability is depicted in the results shown in Figures 4, 5. Furthermore, the analysis performed on the estimates from the two connectivity measures (tdGCMI and tdCC) suggests that STs belonging in different clusters correspond to distinct connectivity patters (e.g., positive or negative correlation). This observation is discussed in more detail in Section 4.4. The exact reasons for having variability on the ST responses to the same input stimulus are not fully understood. However, the findings of this study as well as of other past studies showing this variability lead to the following important conclusion. Many studies use across-trial averaging to study the brain responses. Even though across-trial averaging helps in increasing SNR by eliminating background noise of the recording devices as well as non-time-locked responses to the stimulus, it also distorts and blurs the real ST brain responses. In general, the average smooths out differences between ST responses in terms of duration, timing and intensity of peak activations. The jitter in response is present from the thalamic level. The latency jitter for a strong stimulus like the one used in the experiment we have analyzed is least at the first entry into the cortical circuitry, i.e., at the peaks we see at the EEG and MEG signal at 20 ms, corresponding to activity in BA3b. For both the EEG and MEG peak at 20 ms, corresponding to the first BA3b activation are captured well by the average of 10 single trial with little change in the pattern when all (239) STs are averaged (see Figure 4). The first thalamic response is also captured well by the EEG virtual sensor for P14, with similar peak seen at 15 ms in the averages of 10 and 239 STs, with some improvement in the sharpness of the peak for the average of all 239 STs; this can be explained by either more noise infiltrating the VS output or the presence of rogue STs in the smaller sample of 10 STs. Our analysis of the CMS signal and work by others (Hu et al., 2011) favors the second explanation suggesting the real differences in ST responses around the main peaks produce a mirage of a smooth response that for many people implies a similarly smooth ST response.

### 4.4 Excitation and inhibition from linear and nonlinear connectivity estimates

The normal processing in the brain involves a delicate balance between excitatory and inhibitory influences. Thalamic operations in particular, require inhibitory control across multiple spatial and temporal scales as summarized in a recent review, which highlights two inhibitory systems at work in the thalamus (Halassa and Acsády, 2016). The first, the thalamic reticular nucleus (TRN) sends inhibitory information to the thalamus via inhibitory afferent neural fibers. TRN also controls the magnitude of the cortical input. The second source of inhibitory signals to the thalamus is from subcortical nuclei located outside the thalamus. According to the authors of the study, there is a trend that thalamic inhibition is crucial part of thalamocortical interactions. Furthermore, there is evidence that disturbance of thalamic inhibition can be seen in different disorders. Studying these interactions involving the thalamus, including TRN and subcortical areas are very difficult and until recently could only be performed in animals. DBS has provided opportunities to explore excitation/inhibition in these areas, whenever clinical demands open up a legitimate research investigation. Hanajima (2004) have recorded SEPs using DBS microelectrodes in the region of thalamus of 24 patients in response to median nerve stimulation. Their results suggest that the recorded potentials are generated by excitatory post-synaptic potentials of neurons in the nucleus ventrocaudalis (the human thalamic somatosensory nucleus, Chien et al., 2017). The pathway bringing the excitatory volley on its way to the cortex sends collaterals to the TRN which in turn sends inhibitory influences to the thalamus. Then the thalamus responds to the inhibitory messages and in turn deactivates the excitatory neurons, hence inhibiting the same areas in the cortex, e.g., see Figure 1 in Ferrarelli and Tononi (2010). The novel combination of linear and non-linear connectivity measures we have developed (see Section 2.8) provides a promising way of studying excitation inhibition using simple non-invasive measurements. We discuss the application of the new methodology below. We stress here that a real advance can be made if the results of our analysis can be tested and if necessary improved with experiments using DBS and MEG/EEG measurements on the same subjects (not necessarily in the same experiment, if this is too difficult or demanding for patients).

The analysis of the connectivity results in Section 3.5 gives insights about the nature of the thalamocortical connectivity occurring at 15 ms post stimulation. The grouping of ST-connectivity maps based on the initial clustering of STs (Section 2.6), resulted in separation of the STs with positive and negative correlation values into different clusters (see Figure 7D). This suggests, that there are STs with excitatory influence from the thalamus to the somatosensory cortex, while in other STs an inhibitory influence prevails. At the level of the first connectivity analysis on the entire ensemble of STs (see Section 3.4), the timing and patterns we have identified are consistent with an early excitatory volley from the somatosensory specific thalamus leading to the P20/M20 followed by a second volley from the TRN to the thalamus only a few ms later that could correspond to the deactivation reaching the cortex and appearing as the P30/M30. Alternative pathways also exist which can provide alternative explanations for the origin of the activity and connectivity patterns as we reported in the more detailed analysis of Figure 7, including, as we described above, the inhibitory activity from the thalamus to the cortex, (see panel D, Figure 7, cluster 1). It is indeed remarkable that such thalamocortical inhibitory activity can be probed using simple spatial filtering and clustering on the raw ST EEG/MEG signals.

### 4.5 Advantages and potential applications

The demonstration that VS can be used to extract good estimates of the activity of deep brain sources is one of the important results of this study. The use of VS not only offers a number of advantages against various source reconstruction models, but also against the use of intracranial electrodes. Perhaps the main advantage is that with a priori information about the expected timings of peak activations of certain generators in the brain, the VS can be used to give real time biomarkers about the individual's brain responses. The construction of VS we used is data driven in a way that avoids the modeling assumptions made by source reconstruction models, which face the problem of non-uniqueness of the solution of the inverse problem. Furthermore, because VS are a simple spatial filter they have low demands in processing power and hence, can be performed in real time. A possible real life application would be the use of our methods for the identification of biomarkers in real time recordings comparing healthy individuals with expected patients of demyelination disorders (Hardmeier et al., 2017) and mood disorders (Ferrarelli and Tononi, 2010). Previous studies have already shown that somatosensory evoked potentials are altered in Multiple sclerosis (MS) patients (Hardmeier et al., 2017). Hence, the methods of this study could be applied to better diagnose MS especially in the early stages of the disorder which can therefore allow earlier treatment of the patient greatly increasing efficacy (Waubant, 2012).

### 4.6 Limitations of the study and future work

The methodological concept on which this study is founded on, is the capability of identifying the earliest responses from specific brain areas in the raw signal. These are used to form linear combinations of signals that can act as filters to extract ST activity patterns from the raw signals (EEG or MEG). As the results of this study have shown, the reconstructed ST activation timecourses are reliable estimations (see Section 4.1) of the regional neural activity in the region of the thalamus and the primary somatosensory cortex and can be further used for connectivity analysis between the two regions. However, constructing VS using this concept gives rise to a number of limitations that we have to address for this study to be complete. Firstly, the criteria for selecting the channels are from a single peak in brain activity from averaged ST. Therefore, we cannot make the assumption that the constructed VS is a representation of a specific brain area but rather an approximation of a plethora of areas that are activated at a specific time-slice. Hence, the exact focal localization of the VS in terms of how much spatial accuracy it offers and if it extracts activity from one or many co-activated areas is limited. Yet we have provided evidence that the method is capable of testing assumptions about presence or absence of an activity pattern, estimate connectivity between areas and even explore details of such connectivity. As a minimum the methodology can provide quick review of the measurements and direct further tomographic analysis to a much reduced data sample to analyse. To remove some of these limitations, in future studies the constructed VS will be tested against data from intracranial electrodes measuring the activity from that brain area to determine whether the two signals are correlated. In addition, VS can also be tested against source reconstruction techniques with known high spatial accuracy, for example magnetic field tomography (MFT) (Ioannides et al., 1990, 1995). Another limitation of our methodology is that in order to know the area that the VS is localizing, previous studies that have localized those peaks have to be used. Ergo, this technique is not standalone but it is a low-cost method to reconstruct good estimates of brain regions of interest that have previously been localized and then use these estimations to perform further analysis (e.g., ST connectivity analysis).

## Data availability statement

The data analyzed in this study is subject to the following licenses/restrictions. The datasets presented in this article are not readily available because were provided specifically for the analysis of related to the i-CONN project. Requests to access these datasets should be directed to MA, marios.antonakakis@uni-muenster.de.

## Ethics statement

The studies involving humans were approved by Ethics Committee of the University of Erlangen, Faculty of Medicine on February 20, 2018 (Ref. No. 4453 B). The studies were conducted in accordance with the local legislation and institutional requirements. The participants provided their written informed consent to participate in this study.

## Author contributions

CK: Writing – original draft, Conceptualization, Formal analysis, Methodology, Project administration, Software, Validation, Visualization, Writing – review & editing. MP: Methodology, Writing – review & editing, Formal analysis, Software, Validation, Visualization. VL: Methodology, Writing – review & editing. GO: Writing – review & editing. ST: Writing – review & editing. MA: Writing – review & editing, Investigation. VP: Supervision, Writing – review & editing, Funding acquisition, Methodology. AI: Conceptualization, Formal analysis, Funding acquisition, Methodology, Project administration, Supervision, Validation, Visualization, Writing – review & editing.
