# Supplementary material for: First activity and interactions in thalamus and cortex using raw single-trial EEG and MEG elicited by somatosensory stimulation
Source: Front Syst Neurosci. 2024 Jan 5;17:1305022. doi: 10.3389/fnsys.2023.1305022 (PMC10797085; doi:10.3389/fnsys.2023.1305022)
Supplement: Supplementary file 1 [file Data_Sheet_1.pdf]

Supplementary Material

1 SUPPLEMENTARY TABLES AND FIGURES

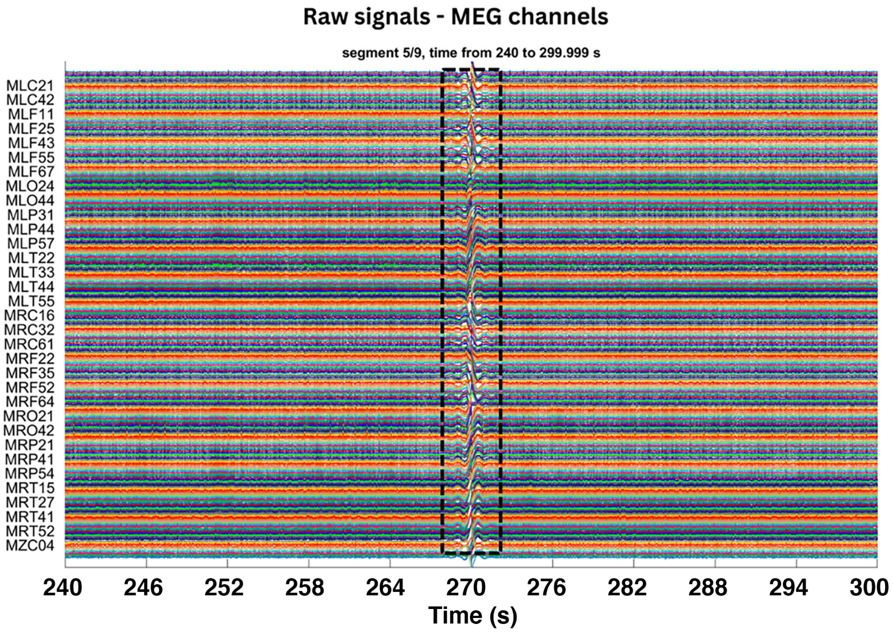

Figure S1. Identification of significant signal distortions by visual inspection of the MEG signals. The vertical plot shows the signal of all MEG channels for a period of $60s$. At the time $270s$, an artifact in all the MEG signals (shown in the black dotted rectangle) is identified as a large and sudden change of the MEG amplitude for all channels. The segment within the rectangle is removed. Further analysis is confined to the segments outside these rectangles.

Table S1. The Table lists, for each subject, the groups of trials, each one between successive periods of large widespread high amplitude events; some of the large events are likely to be movement artifacts. The start and end trial for each group as well as the total number of trials in each group are listed. For each subject, the three groups with the highest number of trials is selected for further analysis (shown in highlight with a different color for each subject, keeping the same color within subject.

Group Number	Subject 01		Subject 02		Subject 03	
	Start – End	# of trials	Start – End	# of trials	Start – End	# of trials
1	1-80	80	**1-176**	**176**	**1-215**	**215**
2	**103-341**	**239**	**188-373**	**186**	229-344	116
3	365-448	84	390-437	48	366-425	60
4	461-590	130	451-538	88	443-498	56
5	**606-747**	**142**	555-600	46	524-593	70
6	760-763	4	617-736	120	**610-748**	**139**
7	776-859	84	**753-887**	**135**	763-859	97
8	**873-1013**	**141**			**883-1178**	**296**
9	1036-1147	112			1197-1198	2
10	1159-1192	34				

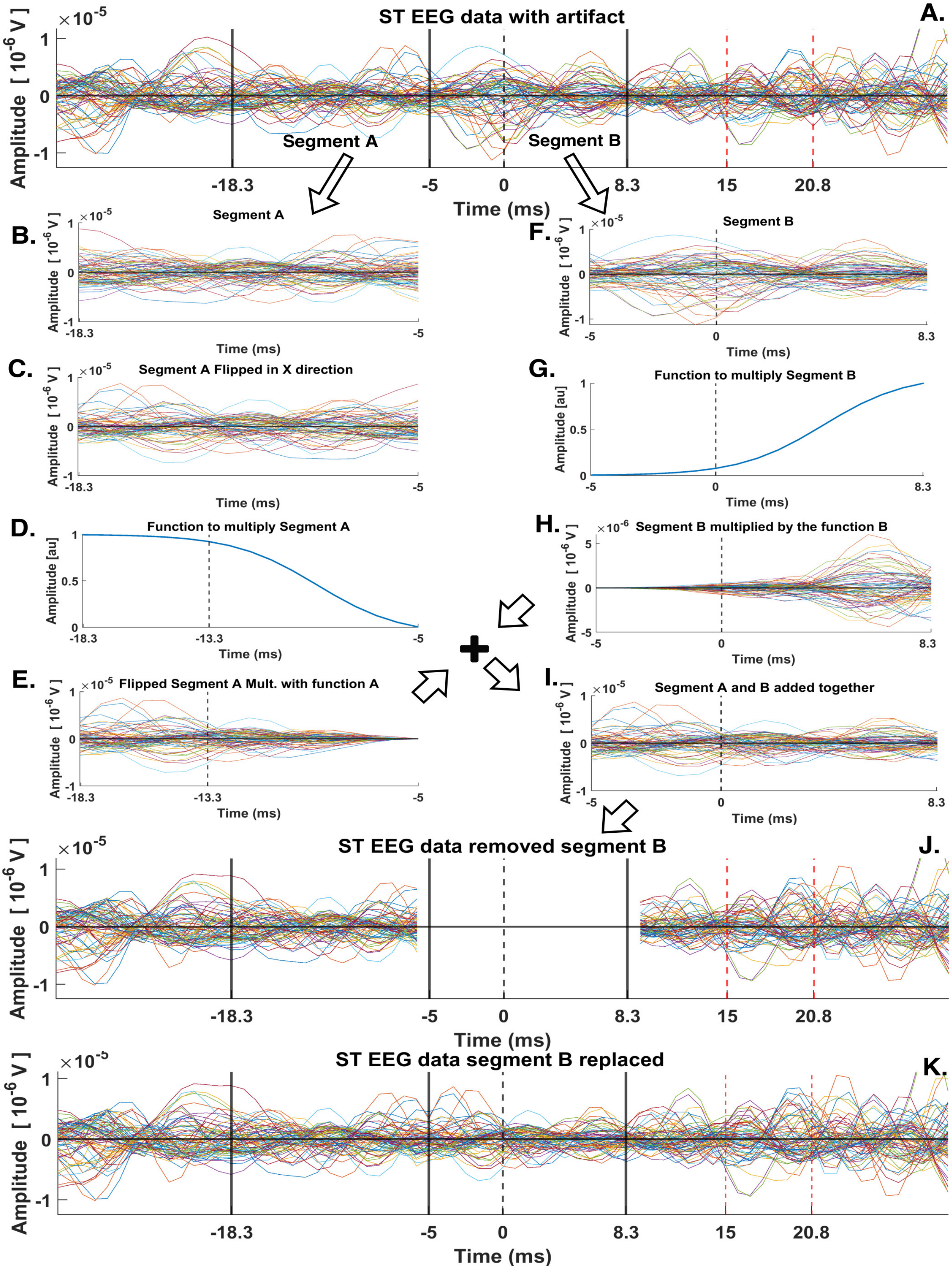

A.
ST EEG data with artifact
Segment A
Segment B
Time (ms)
B.
Segment A
C.
Segment A Flipped in X direction
D.
Function to multiply Segment A
E.
Flipped Segment A Mult. with function A
F.
Segment B
G.
Function to multiply Segment B
H.
Segment B multiplied by the function B
I.
Segment A and B added together
J.
ST EEG data removed segment B
K.
ST EEG data segment B replaced
Amplitude [10-6 V]
Amplitude [au]
-18.3
-13.3
-5
0
8.3
15
20.8

Figure S2. Schematic Diagram showing the methodology used for eliminating the stimulus artifact from the data. Plot A shows the EEG data for 1 random Single Trial (ST). Black solid vertical lines show the two segments A (-18.3 ms to -5 ms) and B (-5 ms to 8.3 ms) that were combined to reduce the stimulus artifact at t = 0 ms (dashed vertical line). Firstly, the segment A (plot B) is flipped (plot C) and then multiplied with a Generalized Logistic Function (GLF), $Y(t) = A + \frac{K-A}{C+e^{\frac{M-t}{B}}}$ with parameters: A = 0, K = 1, C = 0.92, B = 2 ms and M = 10 ms with respect to starting point, as shown in plot D . The segment B (plot F) is also multiplied with the same GLF function and the same parameters except A = 1 and K = 0, as shown in plot G. Then the minimally processed segments A and B, as shown in plots E and H respectively, are added together and the new combined segment is used to replace the original segment B which contained the stimulus artifact at 0 ms. Plot J shows the ST EEG signals with the original segment B removed and replaced with the new combined segment as shown in plot K. In this illustrative diagram, only 1 ST is showed and only for the EEG data, the same procedure is applied for each ST and for both EEG and MEG data. In plots G and D the dash vertical line marks the zero latency point in the replaced signal (which in the original place of each segment corresponded to zero latency for D, but corresponded to -13.3 ms for G). The overall effect of these operations is to keep the properties of the original signal, while reducing considerably the artifact, which now has the same influence at zero as real sources from the brain, while its position is clearly visible in both single STs and average signal and therefore serves as an independent marker of the stimulus onset.

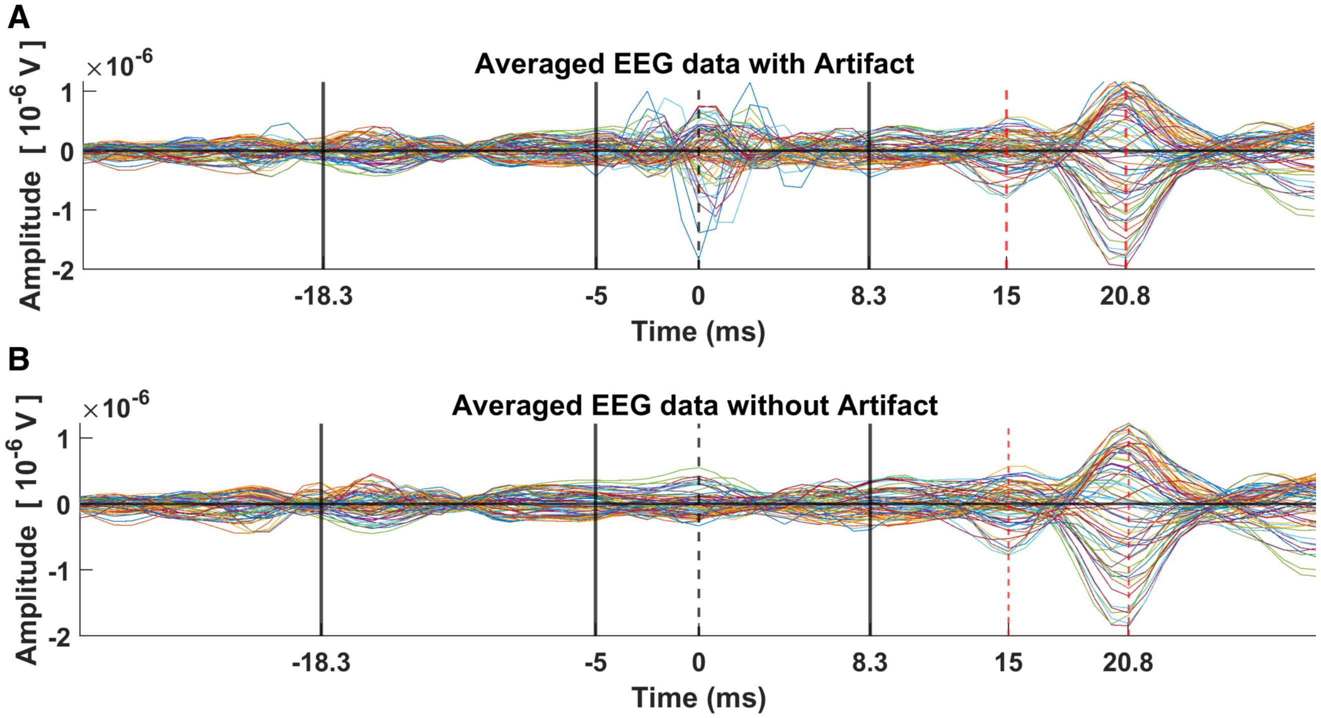

Figure S3. Averaged EEG data before and after applying the stimulus artifact removal method. Plot A shows the average EEG data without stimulus artifact reduction. Plot B shows the averaged EEG data with stimulus artifact reduction. Different line colors are used to show the signals from each different EEG sensor. The dashed vertical line at 0 ms is used to indicate the center time point of the stimulus artifact.

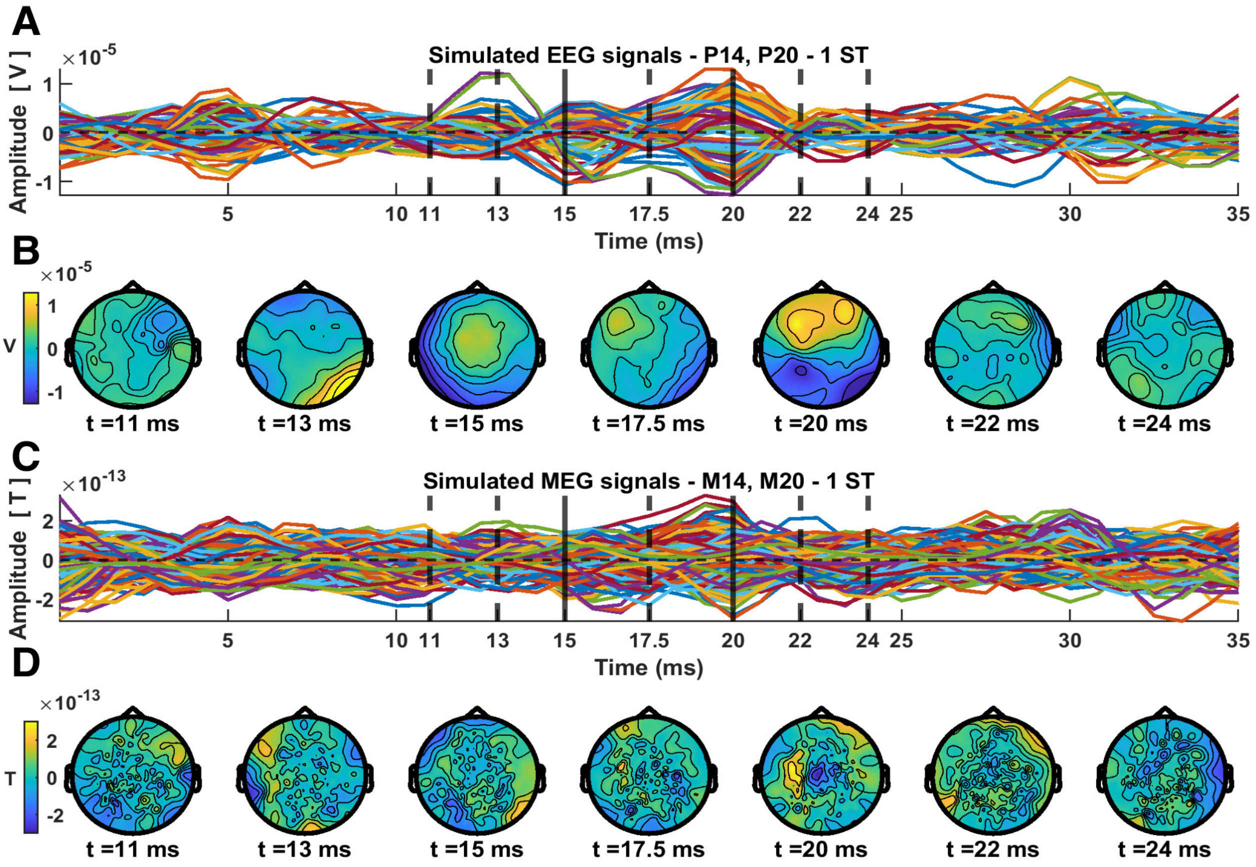

Figure S4. EEG and MEG Composite Model Signals (CMS) for 1 randomly selected Single Trial. The CMS signals of the 71 EEG sensors and the 271 MEG sensors are shown using different colors for each sensor in plots A and C, respectively. The solid lines indicate the times at the peak activation of the thalamic and cortical ECD at 15 ms and 20 ms, respectively, while the dashed lines indicate the times at 5 time points other than the peak activations at 15 ms and 20 ms. The EEG and MEG topographies at the time points indicated by the solid and dashed lines are shown in panels B and D, respectively

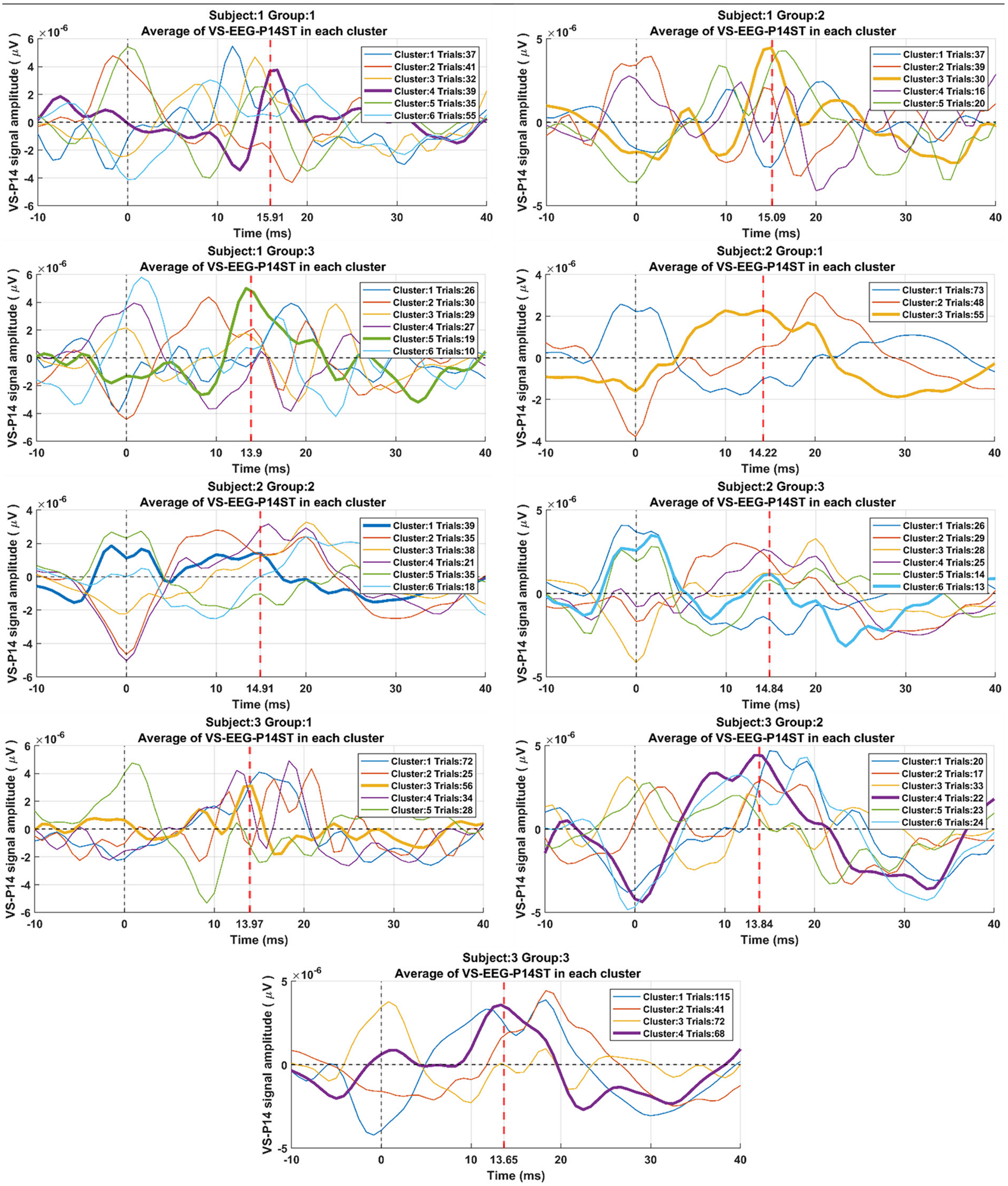

Figure S5. The average signal of clusters of STs extracted by VS-P14. Each plot shows the averages of trials in each one of the clusters in one group of one subject. The one cluster selected for the estimation of the connectivity between the thalamus and the somatosensory cortex.in each plot is shown with a thicker line.

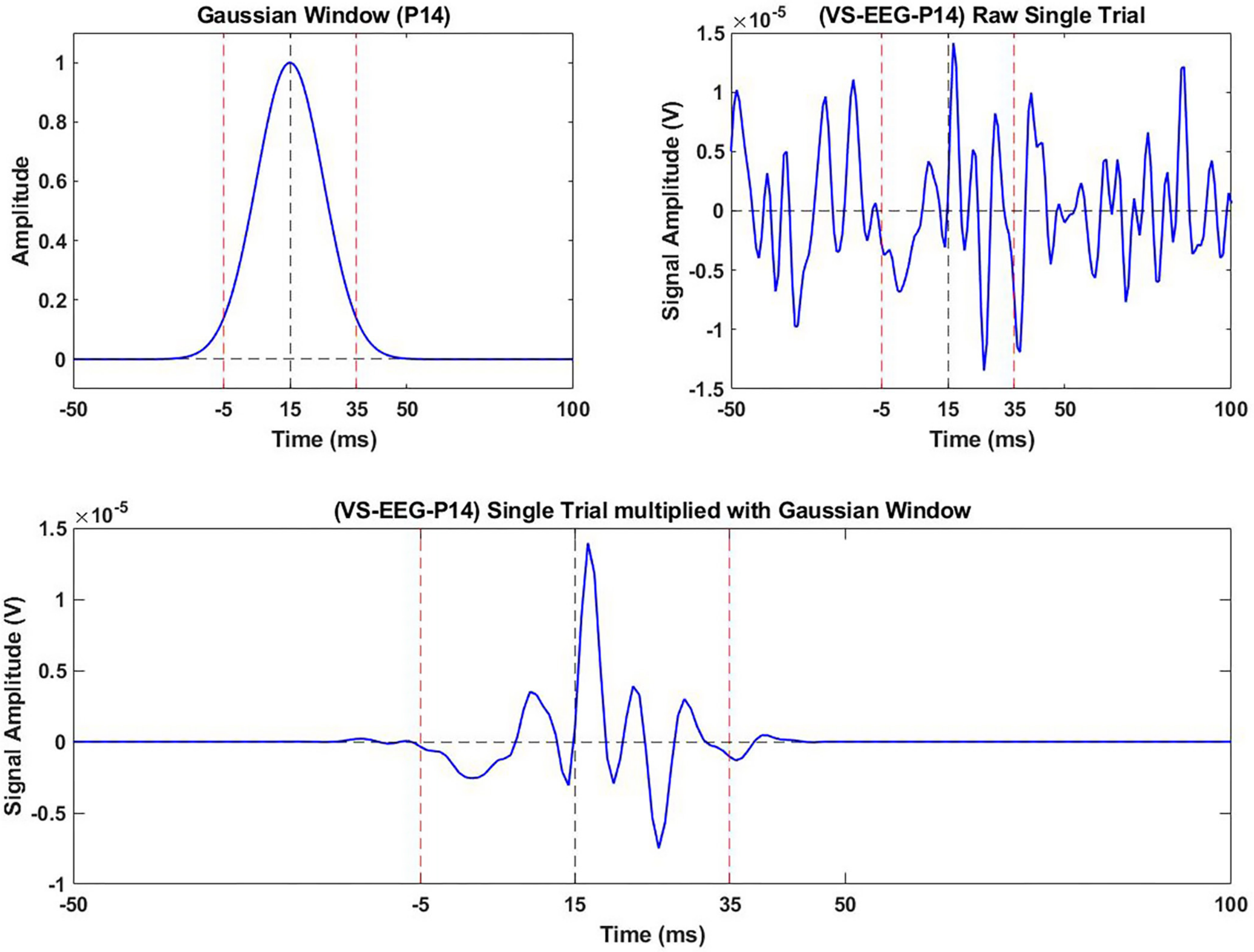

Figure S6. The smooth framing of the estimated timecourse obtained by multiplying with a Gaussian window the output of the Virtual Sensor. The resulting signal in the second row is used for the computation of the correlation between the single trials for the clustering analysis. In the example of a random ST from VS-EEG-P14 the Gaussian window (top left) is centered at 15 ms with a standard deviation σ = 10. The two vertical dot lines show the 2σ in each side where 95% of the signal is contained within 5 ms prior and 35 ms post stimulus onset. Top right plot is the original single trial signal and the bottom plot shows the signal after the multiplication with the Gaussian window.
